# Novel and Selective *Rhipicephalus microplus* Triosephosphate Isomerase Inhibitors with Acaricidal Activity

**DOI:** 10.3390/vetsci5030074

**Published:** 2018-08-23

**Authors:** Luiz Saramago, Helga Gomes, Elena Aguilera, Hugo Cerecetto, Mercedes González, Mauricio Cabrera, Maria Fernanda Alzugaray, Itabajara da Silva Vaz Junior, Rodrigo Nunes da Fonseca, Beatriz Aguirre-López, Nallely Cabrera, Ruy Pérez-Montfort, Alicia Merlino, Jorge Moraes, Guzmán Álvarez

**Affiliations:** 1 Laboratório Integrado de Bioquímica Hatisaburo Masuda, NUPEM-Universidade Federal do Rio de Janeiro campus Macaé and Instituto de Bioquímica Médica, Universidade Federal do Rio de Janeiro, Rio de Janeiro 27971-220, Brazil; saramago@bioqmed.ufrj.br (L.S.); hgomes2@yahoo.com.br (H.G.); rodrigo.nunes.da.fonseca@gmail.com (R.N.d.F.); 2 Grupo de Química Medicinal, Facultad de Ciencias, Universidad de la República, Montevideo 11400, Uruguay; elepao168@gmail.com (E.A.); hcerecetto@gmail.com (H.C.); megonzal@fq.edu.uy (M.G.); 3 Laboratorio de Moléculas Bioactivas-CENUR Litoral Norte, Universidad de la República, Paysandú 60000, Uruguay; mauriciocabreracedres@gmail.com; 4 Centro de Biotecnologia and Faculdade de Veterinária, Universidade Federal do Rio Grande do Sul, Porto Alegre 90040-060, Brazil; mafealga@gmail.com (M.F.A.); itabajara.vaz@ufrgs.br (I.d.S.V.J.); 5 Instituto Nacional de Ciência e Tecnologia-Entomologia Molecular, Rio de Janeiro 22290-180, Brazil; 6 Departamento de Bioquímica y Biología Estructural, Instituto de Fisiología Celular, Universidad Nacional Autónoma de México, CD México 04510, Mexico; bety.aguirre.lopez@gmail.com (B.A.-L.); ncabrera@ifc.unam.mx (N.C.); ruy@ifc.unam.mx (R.P.-M.); 7 Laboratorio de Química Teórica y Computacional, Facultad de Ciencias, Universidad de la República, Montevideo 11400, Uruguay; amerlino@fcien.edu.uy

**Keywords:** triosephosphate isomerase inhibitors, *Rhipicephalus microplus*, acaricidal compounds

## Abstract

The cattle tick *Rhipicephalus microplus* is one of the most important ectoparasites causing significant economic losses for the cattle industry. The major tool of control is reducing the number of ticks, applying acaricides in cattle. However, overuse has led to selection of resistant populations of *R. microplus* to most of these products, some even to more than one active principle. Thus, exploration for new molecules with acaricidal activity in *R. microplus* has become necessary. Triosephosphate isomerase (TIM) is an essential enzyme in *R. microplus* metabolism and could be an interesting target for the development of new methods for tick control. In this work, we screened 227 compounds, from our in-house chemo-library, against TIM from *R. microplus*. Four compounds (**50**, **98**, **14**, and **161**) selectively inhibited this enzyme with IC_50_ values between 25 and 50 μM. They were also able to diminish cellular viability of BME26 embryonic cells by more than 50% at 50 μM. A molecular docking study showed that the compounds bind in different regions of the protein; compound **14** interacts with the dimer interface. Furthermore, compound **14** affected the survival of partially engorged females, fed artificially, using the capillary technique. This molecule is simple, easy to produce, and important biological data—including toxicological information—are available for it. Our results imply a promising role for compound **14** as a prototype for development of a new acaricidal involving selective TIM inhibition.

## 1. Introduction

The cattle tick *Rhipicephalus microplus* is the most significant ectoparasite in tropical and sub-tropical regions, with associated economic losses estimated at US $22–30 billion annually [1]. Its bite causes damage to the skin, promoting local inflammation that harms the animal leather industry. It also affects animal weight gain and, consequently, milk and meat production [2]. It is the most important vector for cattle disease agents, such as *Babesia* spp. and *Anaplasma* spp. [3]. The application of synthetic acaricides is the main method used to control these ectoparasites [4]. However, the continuous and indiscriminate use of acaricides has led to chemico-resistant ticks [5,6]. In addition, these chemicals are polluting the environment and animal byproducts, resulting in new restriction polices for the exportation of these animal byproducts. In several countries, resistance to most acaricides has been confirmed, which represent a worldwide drawback for successful tick control [7,8,9]. *R. microplus* resistance to various acaricides has appeared in chronological sequence, and global spread of resistance to acaricides has become a serious challenge for the livestock industry [10]. Thus, the exploration of new molecules, having novel mechanisms of action with acaricidal activity for *R. microplus*, is still necessary. 

There are some explorations on new molecular targets as antigens for tick control [11]. One example is the observation that RNAi silencing of AamAV422, an *Amblyomma americanum* salivary gland protein, reduces the tick blood meal [12]. Another example is the demonstration that monoclonal antibodies against triosephosphate isomerase (TIM) can inhibit its enzymatic activity and cellular proliferation of the embryonic tick cell line (BME26) [13]. 

TIM is a glycolytic and gluconeogenesis enzyme that catalyzes glyceraldehyde 3-phosphate and dihydroxyacetone phosphate interconversion. Several publications reported the potential of TIM for drug development against various parasites that cause human diseases, such as *Plasmodium falciparum*, *Trypanosoma cruzi*, *Trypanosoma brucei*, *Fasciola hepatica*, and *Giardia lamblia* [14,15,16]. In particular, our group reported the first study using this concept for ectoparasites, and showed that some cysteine residues of *R. microplus* TIM (*Rm*TIM) could be a target for drug design [17]. Although the structural similarity of this enzyme is highly conserved between species, it is possible to obtain selective inhibitors, if they act at the interface of the enzyme, since this part of the enzyme is poorly conserved between species [18]. 

Nowadays, organophosphates are the major chemical acaricides in use (with high environmental impact), which includes pyrethroids, macrocyclic lactones, amidines (like amitraz), fipronil, and fluazuron [19,20]. These can be administered in different forms, such as dip concentrates, spraying, injection, or pour-on treatments [4,21]. Amitraz and Fluazuron were discovered by rational design, in 1973 and 1990 respectively, which are some of the most useful drugs against *R. microplus* [22,23]. Ivermectin (macrocyclic lactones) and some pyrethroids are also very useful drugs, but acaricide resistance threatens their utility [24]. Acaricide discovery is not a very active field of work in academic institutions. From approximately 200 publications in the last 5 years, most of them describe extracts of natural products with acaricidal activity, but with no structural characterization of the constituent compounds [25,26,27,28]. 

By contrast, this work takes a typical medicinal-chemistry approach to find new bioactive molecules with potential acaricidal applications. Based on our experience with parasiticides and enzymatic inhibitors, we screened synthetic molecules from our in-house library, in order to find inhibitors of *Rm*TIM [29,30]. The best inhibitors were identified, and some of these compounds were selected to carry out in vitro assays with *R. microplus* to find new molecules as candidates for future acaricide development. 

## 2. Materials and Methods

### 2.1. Tick Strains 

The Porto Alegre ticks strain (POA) *R. microplus* (Acarina, Ixodidae), were obtained by experimental infestation on bovines (ethical code number 07/2012). This strain was originally collected in the district of Porto Alegre, state of Rio Grande do Sul (Brazil), from a farm without a history of acaricide use and was established in the Faculdade de Veterinária, Universidade Federal do Rio Grande do Sul (UFRGS). It has been maintained under standard laboratory conditions in the absence of acaricide exposure for multiple generations. Ticks are free of parasites, and were reared on bovines obtained from a tick-free area [31]. The partially engorged female ticks were removed from cattle and used for further experiments.

### 2.2. In Silico Analyses 

The DNA sequence analysis, amino acid predictions, and sequence alignments of TIMs from different organisms were performed using the BioEdit (version 7.2.5) [32] and GeneDoc software. (version 5.10) [33]. For phylogenetic analysis, an uprooted neighbor-joining phylogenetic tree was created using the MEGA software (version 6.0) [34]. Bootstrap support was assessed using 1000 replicates. The GenBank accession numbers for TIM used in our analyses were: *Bos taurus* (NP_001013607), *Amblyomma cajennense* (JAC21035); *Amblyomma maculatum* (AEO34689), *Amblyomma parvum* (JAC25576), *Amblyomma triste* (JAC33162), *Rhipicephalus microplus* (ABK76308), *Hyalomma excavatum* (JAP67226), *Ixodes ricinus* (JAA69917), *Ixodes scapularis* (XP_002411305), and *Rhipicephalus pulchellus* (JAA59214). 

### 2.3. Cloning of the RmTIM cDNA in the pET System, Growth of Cells, and Protein Expression 

We amplified the *Rm*TIM open read frame sequence using a plasmid construction obtained by Moraes et al. 2011 as a template for subsequent cloning in the pET3a-HisTEVp plasmid [35]. The 750 bp PCR fragment was generated with the primers forward (FOR) (GGGAATTCCATATGGCCGCAC) and reverse (REV) (CGGGATCCGCTAACCCTGCA), subsequently digested with *Nde*I and *Bam*HI, purified, and cloned directionally in the pET-3a-HisTEVp plasmid. Nucleotide sequence analysis of the clones was performed to confirm the identity of the amplicon and the inserted reading frame. For expression, BL21 CodonPlus *E. coli* cells were transformed with the pET3a-HisTEVp-*Rm*TIM plasmid. Transformed cells were grown overnight at 37 °C in Terrific Broth supplemented with 100 μg/mL ampicillin and 34 μg/mL chloramphenicol. When cultures reached an A600 of 0.6–0.8, 1 mM isopropyl α-D-thiogalactopyranoside was added, and cells were incubated overnight for the expression of TIM.

### 2.4. Purification of RmTIM 

Cells were harvested by centrifugation and suspended in lysis buffer containing 50 mM NaH_2_PO_4_, 300 mM NaCl, and 10 mM imidazole, pH 8.0. Cells were maintained at 4 °C, disrupted by sonication and centrifuged at 100,000× *g* for 1 h. The supernatant was added to a 5 mL HisTrap HP column (GE Healthcare, Chicago, IL, USA) and eluted with an imidazole gradient (0–1000 mM). The eluted protein was added to a HiPrep 26/10 Desalting column (GE Healthcare, Piscataway, NJ, USA) in 50 mM Tris HCl, 0.5 mM ethylenediaminetetraacetic acid (EDTA), 1 mM dithiothreitol (DTT), pH 8.0 (a buffer favoring Tobacco Etch Virus protease (TEVp) activity). The eluted enzyme was incubated overnight at 30 °C with TEVp at a ratio of 20 *Rm*TIM/1 TEVp (*w*/*w*). The digested proteins were then added to a Hi Prep 26/10 Desalting column (GE Healthcare) in a buffer containing 50 mM NaH_2_PO_4_, 300 mM NaCl, and 10 mM imidazole, pH 8.0, and the eluted sample separated in the HisTrap HP column (GE Healthcare). This last step removes the (His)6-tag-TEVpV from the *Rm*TIM. The enzyme was concentrated to 6 mg/mL with Amicon Ultra filters with 30,000 MWCO (Millipore Co., Bedford, MA, USA), and dialyzed overnight in 100 mM triethanolamine (TEA), 1 mM EDTA, and 1 mM DTT. The purification steps were monitored by SDS-PAGE gels containing 16% acrylamide, and stained with Coomassie blue.

### 2.5. Homo sapiens TIM and Rabbit TIM 

*Homo sapiens* TIM (*Hs*TIM) was expressed in *E. coli*, and purified as described in the literature [35]. Rabbit TIM (*Rb*TIM) was obtained commercially from Sigma-Aldrich (Saint Louis, MO, USA). Both enzymes were used in further experiments to test specificity. For inhibition experiments, enzymes were dialyzed extensively against 100 mM triethanolamine and 10 mM EDTA (pH 7.4). Protein concentrations were determined from their absorbance at 280 nm using an extinction coefficient of 34,950 M^−1^ cm^−1^, calculated according to the method of Pace et al. [36].

### 2.6. Triosephosphate Isomerase Activity Assays

TIM activity was determined by measuring the amount of d-glyceraldehyde 3-phosphate converted to dihydroxyacetone phosphate. The reaction mixture contained purified *Rm*TIM (6 ng of protein) in 100 mM TEA, 10 mM EDTA, 1 mM d-glyceraldehyde 3-phosphate, 0.2 mM NADH, and 0.9 units of α-glycerol phosphate dehydrogenase (pH 7.4). Activity was determined following NADH consumption at 340 nm as a function of time, in a HP8452 spectrophotometer (Agilent Technologies, Palo Alto, CA, USA) with a multicell attachment, with a controlled temperature of 25 °C. The assay was made in a traditional cuvette with a final volume of 1000 μL which contained 927 μL of TE Buffer, 50 μL of d-glyceraldeyde-3-phosphate (20 mM), 20 μL of α-glycerol phosphate dehydrogenase (45 U/mL), 2 μL of NADH (100 mM), and 1 μL of TIM (6 mg/mL) [17].

### 2.7. Inhibition Screening and Compound Library

The inhibition was carried out under the same conditions as the activity assays. Before the determination of the activity, the selected compounds and TIM were incubated at 25 °C for 2 h. The selected compound was dissolved in DMSO and diluted to 10% (*v*/*v*) of DMSO in the incubation mixture. The 227 molecules were chosen from an in-house library, with diverse chemical structures. Based on sequence similarities found in the alignment of different TIMs, in particular, between the TIMs from *T. cruzi* and *R. microplus*, we chose molecules that had previously shown either antiparasitic activity or inhibition of TIM [29,30]. In a first round of screening, the compounds were assayed at 100 µM. A compound was considered active if the enzymatic inhibition was higher than 50%. The active compound was assayed at different concentrations to determine the IC_50_ value. The IC_50_ was defined as the drug concentration at which 50% of the initial velocity was determined, relative to the control (no drug added), and analyzed using OriginLab8.5^®^ sigmoidal regression (% of enzymatic activity vs logarithm of the compound concentration). All assays were done in triplicate, and the average error for each measurement did not exceed 10%. 

### 2.8. Ligand–Protein Molecular Docking 

The geometrical structures of compounds **50**, **98**, and **14** were fully optimized in aqueous solution at the PM6 semi-empirical level using the IEF-PCM continuum model with Bondi atomic radii. For molecular docking studies, the crystallographic structure of *Rm*TIM (PDB ID 3TH6) was used [17]. Docking calculations were carried out with Autodock 4.2 using the implemented empirical free energy function and the Lamarckian genetic algorithm. The AutoDockTools package was employed to generate the docking input files and to analyze the docking results. Gasteiger–Marsili charges were used for both proteins and ligands. Since the location of the compounds in the enzyme was unknown, a grid map with 124 × 126 × 126 points and a grid-point spacing of 0.6 Å was applied in order to explore the entire protein surface. The maps were centered on the macromolecule. Each docking consisted of 50 independent runs, with an initial population of 150 individuals, a maximum number of 2.5 × 10^5^ energy evaluations, and a maximum number of 27,000 generations. Default values were used for the remaining parameters. Results differing by less than 2.0 Å in root-square deviation were grouped into the same cluster. 

### 2.9. MTT Viability Assay 

*R. microplus* embryonic cell line BME26 suspension was seeded into 24-well plates (5 × 10^5^ cells/well) to a final volume of 500 μL in complete medium, and allowed to attach. After 24 h incubation at 34 °C, chemical inhibitors were added at the final concentrations indicated, and 0.05% DMSO was used in negative control wells. After 24 h of treatment, 50 μL MTT prepared in serum-free medium (5 mg/mL) was added to each well. Media were completely discarded after 2 additional hours of incubation, and 1 mL of acid-isopropyl alcohol (0.15% HCl in isopropyl alcohol) was added to dissolve the formazan crystals. The mixture was transferred to 1.5 mL tubes, centrifuged at 6000× *g* for 15 min, and the clear supernatant collected in new tubes for absorbance measurement at 570 nm using quartz cuvettes in an UVmini-1240 UV–vis spectrophotometer (Shimadzu, Japan). Unless otherwise stated, the absorbance values of the control treatment were used for normalization (100% viability) [37].

### 2.10. Effect of Compounds on BME26 Cell Cultures 

The *Rhipicephalus microplus* embryonic cell line BME26 was maintained in Leibovitz’s culture medium supplemented with amino acids, glucose, mineral salts, and vitamins [38]. For the tests, BME26 cells were adjusted to a concentration of 10^6^/mL, and aliquots of 0.1 mL were added to 24-well microtiter plates and incubated for 24 h at 34 °C. Then cells were incubated for 24 h with 25, 50, or 100 µM of compounds **50**, **98**, and **14.**

### 2.11. Effect of Compounds on BME26 Morphology 

BME26 cells (5 × 10^5^ cells/well) were plated on glass coverslips (Corning^®^ Costar^®^, Cambridge, MA, USA) in 24-well plates, and incubated in complete medium to attach at 34 °C for 24 h. Chemical inhibitors were added at the final concentrations indicated, and 0.05% DMSO was used in negative control wells. After 24 h of treatment, the cells were washed with 0.15 M NaCl, 10 mM sodium phosphate, pH 7.2 (PBS), and immediately fixed on a buffered (PBS) formaldehyde 4% solution for 15 min at room temperature (RT). The cells were then incubated for 20 min (RT) with 200 µL of a solution containing the nuclear marker DAPI (4′,6-diamidino-2-phenylindole, dihydrochloride, molecular Probes, D1306-1 µg/mL), and 1 μL of the F-actin probe phalloidin (Alexa Fluor^®^ 555, Molecular Probes, A34055-300 units). The cells were visualized using a Leica DMI4000 inverted fluorescent microscope equipped with two A4 (DAPI) filter cubes and N2.1 (Phalloidin). Pictures were obtained with a DFC365 FX camera, and images were mounted into a single file with the help of Adobe Photoshop (CC19.0).

### 2.12. Adults Immersion Test

Each group of 15 fully engorged females was immersed in 50 mL tubes containing 40 mL of compound **14** at different concentrations (500 and 2000 ppm) for five minutes. Two negative control groups were used, one in PBS and another in DMSO 10%. After immersion, the fully engorged females were dried on absorbent paper. They were then placed in 100 mm disposable Petri dishes, identified, and taken to the Bio-Oxygen Demand chamber, where they were kept at 27 °C ± 1 °C and relative humidity of 80% ± 5% for 21 days. Females that did not lay eggs were considered dead. The eggs were weighed and transferred to disposable plastic syringes closed with hydrophilic cotton and returned to the Bio-Oxygen Demand under the same conditions, until the larvae had hatched. The efficacy of compound on *R. microplus* physiology was calculated based on the weight of the fully engorged females, eggs, larvae, and percentage of hatching, according to Drummond et al. 1973 [39]. The statistical analyses were performed using one-way ANOVA followed by Tukey’s test. *p* values around 0.05 were considered statistically significant.

### 2.13. Effect of Administration of Inhibitor ***14*** in Partially Engorged Female Ticks

Partially engorged female ticks (obtained as described in Section 2.12) after 30 days of feeding, weighing 30–60 mg, were collected from bovines and fixed with double-sided tape in a polystyrene surface, and fed with bovine blood by capillarity for 28 h [40,41,42]. During the feeding process, 2 µL of the compound (at 1 mM in DMSO) was injected in the lower quadrant of the ventral surface, using a Hamilton syringe with a 0.460 mm needle gauge (the control groups were inoculated with DMSO). Female feeding was performed at 28 °C in 85% humidity. All parameters were analyzed after feeding.

### 2.14. Effects of RmTIM Inhibitor ***14*** on Tick Physiology 

The effects of *Rm*TIM inhibitor **14** were evaluated by inoculation and immersion tests. The ticks were handled as described in Section 2.12 and Section 2.13. The parameters analyzed after that were total egg weight, total larval weight, eclosion rate, and mortality (percentage of dead females). For these terms, the females that did not lay eggs were considered dead. All statistical analyses were performed using one-way ANOVA followed by Tukey’s test. *p* values around 0.05 were considered statistically significant. 

### 2.15. Oral Acute Toxicology in Mice 

The in vivo LD_50_ for compound **14** was determined following the guidelines described by Organization for Economic Co-operation and Development O.E.C.D. [43]. Briefly, healthy young adult male mice (strain B6D2F1, 60 days old, 25–30 g) were used in this study. Initially, the compounds used in our study were administered at a dose of 2000 mg/kg, by means of an orogastric canula, to a first animal. This animal was fasted, maintained, and observed during 14 days. Depending on the results after 48 h, another animal received a new dose, if the original animal survived these 48 h. If not, the dose was reduced to ¼ of the initial dose and administered, and the new animal was included in the test. The procedure was repeated until the LD_50_ was determined. Every experimental protocol with animal were previously approved by local ethics committee in animal experimentation [44].

## 3. Results

### 3.1. Sequence Analyses

**Figure 1:**
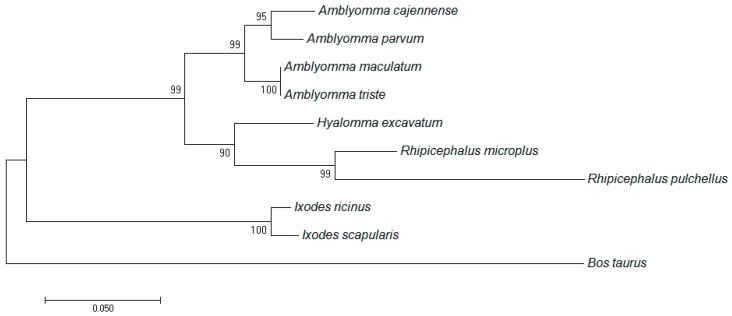
Molecular phylogenetic analysis of triosephosphate isomerase (TIM) sequences. The evolutionary history was inferred by using the maximum likelihood method. Sequences from different tick species and also the *Rhipicephalus microplus*-specific host (*Bos Taurus*) were included in the analysis.

The deduced amino acid sequence of *R. microplus* TIM had high similarity with other tick and *Bos taurus* TIM sequences. Interestingly, remarkable differences in the number of cysteine residues were observed. There are five cysteine residues that occur in almost all TIMs from ticks, but are not present in *B. taurus* TIM (natural host for *R. microplus*) (Figure S1 in supporting information). A phylogenetic analysis of TIMs from ticks was constructed based on their amino acid sequences (Figure 1). The phylogenetic tree revealed two clades for the tick enzymes: one clade includes *R. microplus* and *Amblyomma* spp. TIMs, and another clade formed by *Ixodes* spp. TIMs. As expected, *B. taurus* TIM belongs to a well-separated clade. 

### 3.2. Chemical Structure Leads Used in a Primary In Vitro Screening

**Figure 2:**
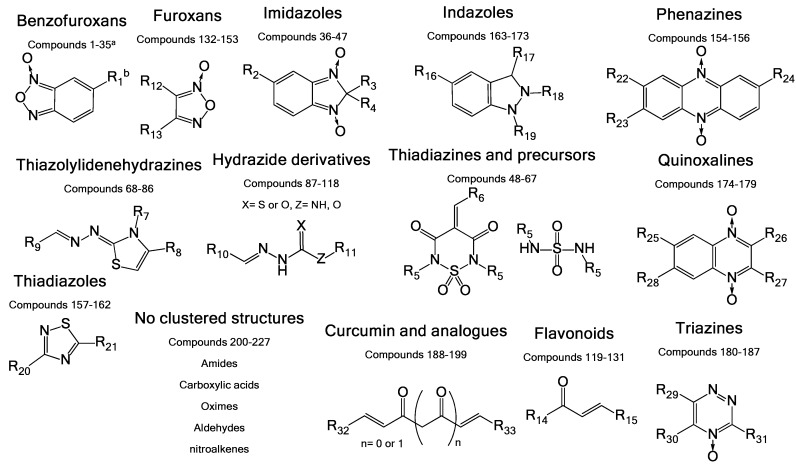
General structures of the different chemotypes studied as *R. microplus* TIM (*Rm*TIM) inhibitors. We chose 227 molecules with diverse chemotypes from different families of compounds including benzofuroxans, benzimidazoles, thiadiazines, hydrazide derivatives, thiazolylidenehydrazines, flavonoids, furoxans, phenazines, thiadiazoles, indazoles, quinoxalines, triazines, as well as other diverse structures. ^a^ Compounds are numbered according to Table S1 shown in the Supplementary Material. ^b^ R groups correspond to different structural moieties.

The molecules used in this work were chosen from an in-house chemo-library of a thousand compounds that have a structure–activity relationship related to trypanosomicidal and TIM-inhibition activities. Two hundred and twenty seven compounds were selected based on their previously reported activity against other parasites. We chose molecules with diverse chemical structures from different families of compounds, including benzofuroxans, benzimidazoles, thiadiazines, hydrazide derivatives, thiazolylidenehydrazines, flavonoids, furoxans, phenazines, thiadiazoles, indazoles, quinoxalines, and triazines, among others (Figure 2).

### 3.3. In Vitro Inhibition at Recombinant Enzymes

**Figure 3:**
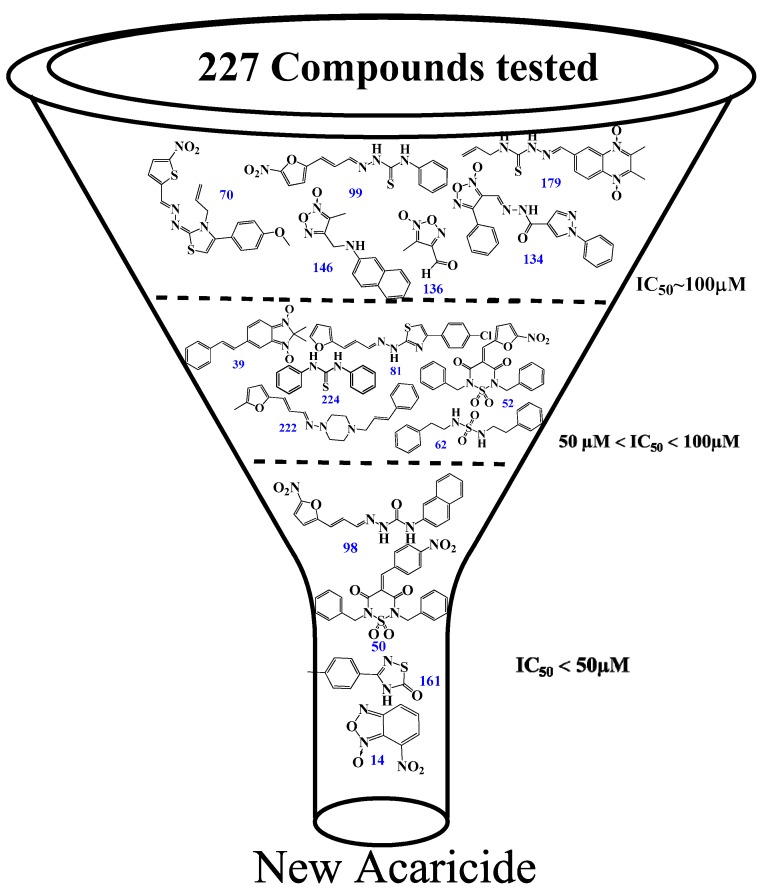
Inhibition results on *Rm*TIM. Structures of the best *Rm*TIM inhibitors are identified herein. The compounds are numbered according to Table S1 (see Supplementary Material section). We found a variety of compounds with activity at doses between 100 and 25 µM, although most of the families were inactive. Three zones of inhibitory activity were identified: IC_50_ ~ 100 µM (low activity), IC_50_ between 50 and 100 µM (moderate activity), and IC_50_ ˂ 50 µM (good activity).

Initially, a primary in vitro screening with 227 compounds at a concentration of 100 µM was performed to find specific *Rm*TIM inhibitors (all of these results are in Table S1). Sixteen new hits were identified as new candidates to become inhibitors (an inhibition of more than 50% of *Rm*TIM activity at 100 µM). We observed that 7% of the compounds tested were active. Compounds **70**, **99**, **134**, **136**, **146**, and **179** were poorly active (IC_50_ ~ 100 µM). Compounds **39**, **52**, **62**, **81**, **222**, and **224** had medium activity (IC_50_ between 50 µM and 100 µM). Four compounds showed higher ranges of inhibition (IC_50_ lower than 50 μM). These were compounds **14** (IC_50_ = 49 µM), **50** IC_50_ = 50 µM), **98** IC_50_ = 50 µM), and **161** (IC_50_ = 25 µM). Subsequently, it was confirmed that these compounds had no considerable inhibition activity for mammalian TIMs (rabbit and human) (Table 1 and Figure 3).

### 3.4. Molecular Docking Simulation Analysis

**Figure 4:**
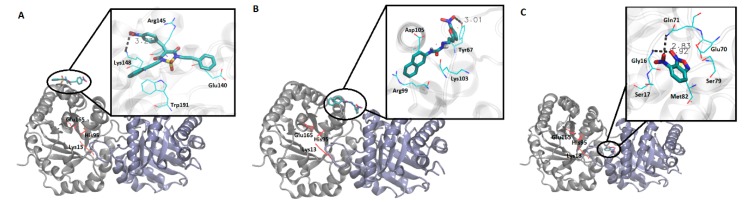
Molecular docking studies for compounds **50**, **98**, and **14**. Graphic representation of the interaction between each inhibitor with *Rm*TIM. (**A**) Compound **50**, amino acids from the active site are depicted in red. The zoom in the inset shows ligand–enzyme interactions at the binding site. (**B**) Compound **98**, amino acids from the active site are depicted in red. The zoom in the inset shows ligand–enzyme interactions at the binding site. (**C**) Compound **14**, amino acids from the active site are depicted in red. The zoom in the inset shows ligand–enzyme interactions at the binding site.

Docking studies of compounds **50**, **98**, **161**, and **14** contributed to elucidating how they interact with residues from different regions of *Rm*TIM. It was observed that compound **50** interacts with amino acids located in alpha-helix 5 (Arg145, Lys148, and Glu140) (Figure 4A). Compound **98** interacts with amino acids of alpha-helix 4 (Arg99, Lys103, Tyr67, and Asp105) (Figure 4B). Compound **161** was located very near the active site (in a conserved zone) (data not shown) and Compound **14** interacts with interface residues present in the loop 3 (Glu70, Gln71, Ser79, and Met82), and alpha-helix 1 (Gly16 and Ser17) (Figure 4C).

### 3.5. In Vitro Inhibition of the BME26 Cell Line 

**Figure 5:**
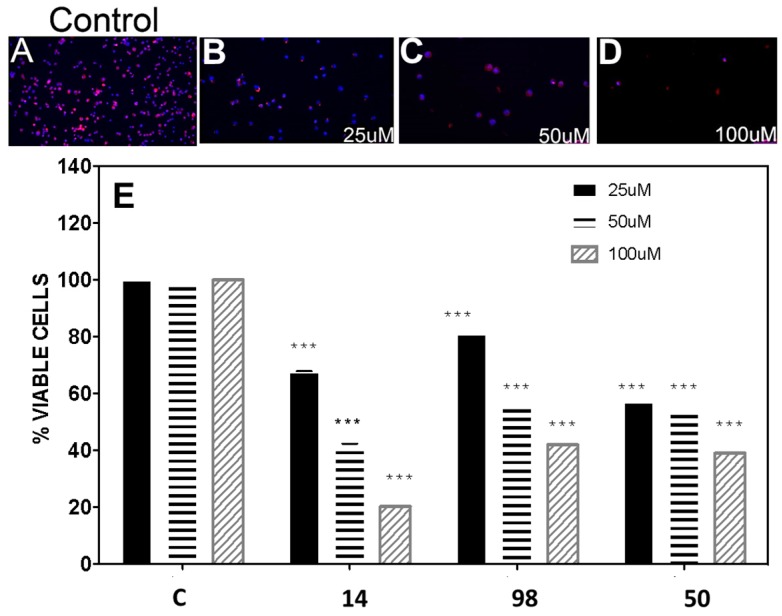
In vitro activity of selected compounds on BME26 embryonic cells from *Rhipicephalus microplus*. (**A**–**D**) Confocal fluorescent microscopy of BME26 cells incubated for 24 h with different concentrations of compound **14**. Following incubation, cells were stained with DAPI and phalloidin to observe the cell architecture (×100 magnification). (**A**) Control cells, (**B**) cells incubated with 25 µM of compound **14**, (**C**) cells incubated with 50 µM of compound **14**, (**D**) cells incubated with 100 µM of compound **14**. (**E**) Dose–response effects for compounds **14**, **98**, and **50** in the MTT assay; C is the control without treatment (0.1% (*v*/*v*) DMSO).

The inhibitors **14**, **50**, **98**, and **161**, were assayed in the embryonic cell line BME26 using the MTT assay (Figure 5). It was observed that cell viability at 100 µM was 20%, 42%, 40%, and 100% for compounds **14**, **50**, **98**, and **161,** respectively. The IC_50_ values for the active compounds were 38 ± 4 µM (**14**), 71 ± 8 µM (**50**), and 67 ± 4 µM (**98**), respectively. Inhibitor **161** did not have any effect. 

### 3.6. Effect of TIM Inhibitor ***14*** on the Physiology of the Tick 

The effect of TIM inhibitor **14** on fully engorged females was analyzed by an adult immersion test. When compared to vehicle compound, **14** was able to reduce the eclosion rate by 16% at different concentrations (500 ppm and 2000 ppm) (Table 2). The effect on partially engorged females was analyzed by injection of compound **14** into females treated during the artificial feeding procedure. This resulted in 0% survival, when compared with a negative control. Consequently, no egg hatching was observed (Table 3).

### 3.7. Effect of TIM Inhibitor in Oral Acute Toxicology in Mice

The LD_50_ value of the compound **14** for mammalian was 600 g/kg body weight. This is considered a slight toxicity according to the World Health Organization guidelines. 

## 4. Discussion 

An important source for the discovery of new drugs is the in vitro screening of chemical libraries using a validated target. In vitro screening has been very useful in many areas of health, such as cancer, cardiovascular and kidney diseases, infection control of many pathogens (viruses, parasites, bacteria, and fungus) [45,46,47,48]. One of the criteria for the election of a molecular target is selectivity. A selected target, to fight a pathogen, needs to be different to the host biomolecules. The deduced amino acid sequence of *Rm*TIM had high similarity with other sequences from TIMs of other ticks and *Bos taurus*. Interestingly, different numbers of cysteine residues were observed in the sequences. There were five cysteine residues that are present in almost all tick TIMs, but are not present in *B. taurus* (the natural host of *R. microplus*) (Figure S1 in supporting information). A phylogenetic analysis of different tick TIMs was constructed using the amino acid sequences studied (Figure 1). The phylogenetic tree revealed two clades: one clade included *R. microplus* and *Amblyomma* spp. TIMs, and the other was formed by *Ixodes* spp. As expected, *B. taurus* TIM segregated into a well-separated clade. The major differences among these sequences are in the dimer interface [49], which gives the possibility to find a selective inhibitor that interacts with the dimer interface [29,50]. TIM from *B. taurus* is not yet available for use in inhibition assays, but the structurally very similar TIM from rabbit is (*Rb*TIM). We used *Rb*TIM for the selectivity studies [51,52].

In this work, an initial screening was performed using 227 compounds from an in-house chemo-library, selected according to previous reports of their activity against other parasites [15,53,54,55]. The choice was based on chemical structures that included different families of compounds, which were benzofuroxans, benzimidazoles, thiadiazines, hydrazide derivatives, thiazolylidene hydrazines, flavonoids, furoxans, phenazines, thiadiazoles, indazoles, quinoxalines, and triazines, among others (Figure 2A). Until the present date, no molecule with any of these chemical structures, or another structure, has been reported to be a specific inhibitor of *Rm*TIM. 

The inhibitory capacity of these 227 compounds was tested in vitro against RmTIM using an initial concentration of 100 µM. Under these conditions, 50% inhibition of *Rm*TIM activity was defined as an arbitrary cut-off point in order to define the molecules that were to be considered for more detailed studies. Based on this criterion (50% inhibition of *Rm*TIM activity at 100 µM, Table S1) only some compounds belonging to some families were active, and the thiadiazines and furoxans showed the highest scores (±15% of these molecules were active). Among the hydrazide derivatives, 9% of the molecules were active, and within the other families, only 3% of the evaluated compounds displayed *Rm*TIM inhibitory activity. Based on this criterion, sixteen compounds from all the chemotypes (7% of the tested molecules, Figure 3) were selected for the study of their inhibitory capacity at different concentrations, in order to determine the IC_50_ values. In addition, the selectivity of the four compounds with highest inhibition capacity compound **98** (hydrazide family), **50** (thiadiazine family), **161** (thiadiazole family), and **14** (benzofuroxan family)) (Table 1) against *Rm*TIM were assayed by comparing their performance against *Hs*TIM and *Rb*TIM, which has a sequence identity of 98% with *B. taurus* TIM.

Interestingly, the inhibitory activity of the compounds against *Rm*TIM shows significantly lower values when compared with the IC_50_ previously obtained for *Trypanosoma cruzi* TIM (*Tc*TIM), indicating higher potency as inhibitors. Compound **161** was the most active against *Rm*TIM, although it also displayed a slight inhibitory activity against *Hs*TIM at 100 µM (Table 1). Also, this compound had an IC_50_ of 4 µM for *Tc*TIM vs the 25 µM in *Rm*TIM. Compounds **98**, **50**, and **14** showed medium inhibitory activity (around 50 µM for the IC_50_). On the other hand, they did not affect the mammalian TIMs, showing a desirable selectivity profile. Compounds **161** and **50** have more complex synthetic procedures, while compounds **98** and **14** are simpler to prepare. In addition, these last two compounds support structural modifications, which are important for future improvement in molecular synthesis for obtaining a better biological activity. In particular, a wide spectrum of structural modifications was included in the initial screening for the thiadiazine family, but only the arrangement of compound **50** was successful, suggesting that a future modification to obtain a more active molecule with easy and low-cost synthetic procedures cannot be possible. 

It is important to highlight that these four molecules are the first inhibitors reported, until now, for *Rm*TIM. Usually, it is expected the inhibition range may be lower than 10 µM, however, the search for species-specific drugs could vary according to various parameters, including the target, the disease, model organisms, and pharmaceutical formulation [56,57,58]. Nonetheless, there are few manuscripts on drug design against others arthropods to compare the potency of the candidate compounds identified [59,60,61,62]. It is significant to observe that these four molecules are selective molecules, since they have a negligible effect on mammalian TIMs, even when used at a 4-fold higher dose than their IC_50_ for *Rm*TIM. Furthermore, these are lead compounds which can be improved with further chemical modifications [63,64]. 

Energy metabolism is essential during embryogenesis of the cattle tick. Marker enzymes of metabolic pathways, such as hexokinase and pyruvate kinase, show high levels, before formation of the cellular blastoderm [65]. Enzymes of the gluconeogenesis pathway have more activity after this point, and are responsible for glycogen accumulation that can be used as an energy source, until the eclosion of the embryos [65]. TIM is an enzyme involved in both processes that could act in different metabolic situations [13]. Therefore, these molecules represent promising lead compounds in the search of effective drugs against these parasites, and could potentially be developed as novel acaricides. Also, these structures are very different to the commercial acaricides, and this characteristic can be useful to bypass the resistance observed in different field populations of ticks [5,66]. 

In order to explore the mode of binding of compounds **50**, **98**, and **14** to *Rm*TIM, molecular docking experiments were performed, mapping the entire protein surface of the dimer. Compound **50** binds on the surface of the enzyme, establishing a weak hydrogen bond (3.2 Å) between one oxygen atom of the nitro moiety of the ligand and the Να nitrogen of Lys148, and also has π–cation and π–anion interactions with Arg145 and Glu140, respectively (Figure 4A). The binding mode of this compound could explain its lower activity since, being weakly bound to the surface of the protein and taking into account the high flexibility of this kind of enzyme [67,68], it could be prone to expulsion into the solvent by the normal dynamics of *Rm*TIM. Compound **98** also binds superficially to *Rm*TIM, forming a hydrogen bond of 3.0 Å with Tyr67, and makes contacts with the amino acids that form alpha-helix 4, such as Arg99, Lys103, and Asp105 (Figure 4B). 

Compound **14** binds to the dimer interface interacting with residues on loop 3 (Glu70, Gln71, Ser79, and Met82) and on alpha-helix 1 (Gly16 and Ser17) (Figure 4C). This compound establishes two strong hydrogen bonds with Gln71 from chain A and Gly16 from chain B (2.8 Å and 2.9 Å, respectively), a fact that would contribute to its stabilization within the binding site. The location of this compound near loop 3 is interesting, considering its inhibitory potency, since residues in this loop are involved in hydrogen bond interactions with loop 1 of the other subunit, helping to maintain the integrity of the dimer. According to these docking results, this compound could be acting through the perturbation of the interface region, leading to the disruption of the dimer, as has been already reported for other TIM inhibitors [14,16,69]. Moreover, this region of the protein is less conserved among TIMs, a fact that would explain the high selectivity of compound **14** against *Rm*TIM.

To improve and complement the characterization, the selected inhibitors, **14**, **50**, **98**, and **161**, were assayed with the *R. microplus* embryonic cell line BME26 using the MTT assay (Figure 5 and Figure S2). Compound **161** showed no activity at a concentration of 100 µM in the assayed conditions. Although this compound was the best inhibitor against *Rm*TIM and *Tc*TIM in the enzyme test, the incapacity to inhibit the TIM activity in BME26 cells can be explained since, in previous work, it was found that compound 161 cannot penetrate biological membranes [53]. The IC_50_ for benzofuroxan **14** was 30 ± 4 µM (tenfold less active than ivermectin (3 µM)) a widely used acaricide [8,42]. Besides the lower activity of compound **14,** compared to ivermectin, this compound is structurally simple and has a lower cost of production. On the other hand, thiadiazine **50** and hydrazide **98** were less active in BME26 cells, with IC_50_ values near 50 µM. 

The detoxification of acaricides occurs due to families of genes involving different enzymes, such as the cytochrome P450 superfamily [70,71]. Methylglyoxal is a glycolytic intermediate, which may reach high levels when TIM levels decrease in the cell. It acts as a highly reactive electrophile that glycates and crosslinks proteins or DNA through the formation of advanced glycation end products [72,73].

The viability of BME26 cells was reduced at high concentrations of compound **14** (Figure 5). This molecule has been shown to have low toxicity for human cells [74] and has no toxic effects on murine cells at a concentration of 100 µM [75]. Besides that, toxicological data are available [76,77,78]. Moreover, this molecule was reported to have agricultural applications as a herbicide and in phytopathogen control [79,80]. In this context, compound **14** is an interesting prototype for future chemical modifications to enhance its acaricidal activity [81]. 

Finally, we performed a proof of concept experiment of the acaricidal potential of compound **14** using the adult immersion test (Table 2). To evaluate a topical application of compound **14**, we test the concentrations of 500 and 2000 ppm, and the results showed a reduction of 16% in the eclosion rate. Similar results were reported with coumaphos, a commercial acaricide, at a concentration of 500 ppm [82]. These results are not promising because of the solubility problem of the compound. Typical immersion tests use aqueous solutions, then this compound was low soluble in this test conditions. If the compound is a solid suspension in the immersion test, there will not be absorption into the animals, and then there are no toxic effects. To resolve this kind of problem, a structural modification of the compound is needed to improve the solubility, to be applicable in a bath on the fields as an agrochemical. Also, could be performed a better formulation for the baths like more lipophilic solution, but this is not useful in the immersion test.

Besides that, partially engorged ticks were injected with 2 nmol/tick of this compound, and fed in an artificial feeding assay [41]. The biological effect was observed by an induced mortality of 100% (Table 3). The acute oral LD_50_ value of the compound **14** was 600 mg/kg body weight, while other well-known acaricides had lower values in mice, such as fipronil (91 mg/kg) [83] and coumaphos (28 mg/kg) [84]. Some difficulties were found with DMSO that was used as a vehicle; the solubility was improved but there is some intrinsic toxicity because of the DMSO, but the effect from the administration of the compound **14** was clearly observed. The mortality was improved compared to DMSO treatment. This result suggests that compound **14** could be a promising acaricide and also a novel scaffold for new acaricide development, with a novel mechanism of action. 

## 5. Conclusions

**Figure 6:**
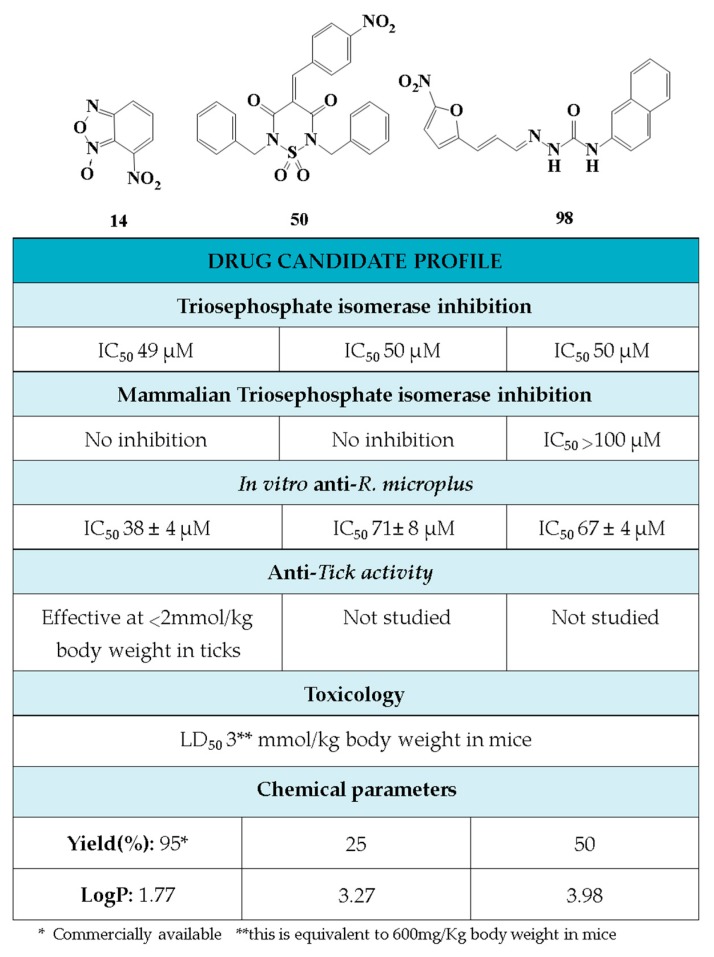
Drug Candidate Profile. An abstract of the pharmacological information compiled in this work for the identified hit compounds **14**, **50**, and **98**.

In conclusion, in this study, we screened 227 compounds, from which four were found to be particularly active and specific inhibitors of *Rm*TIM. Among these, compound **14** (7-nitrobenzo[c][1,2,5]oxadiazole 1-oxide) is a molecule that can be obtained by a simple synthetic procedure, and can be produced on a large scale (Figure 6). Moreover, it shows inhibitory activity against *Rm*TIM, together with an adequate specificity. In addition, this molecule, the first *Rm*TIM inhibitor reported, showed acaricidal activity in biological assays. It is important to highlight that the resistance to commercial acaricides has increased over the years. A molecule with a different structure and mechanism of action, such as compound **14**, is important to bypass the resistance present in field tick populations. Also, this molecule has a low toxicity that is within the limits of the Food and Drug Administration (FDA) for a safe drug. The four molecules identified here are hits for the development of new acaricidal drugs. 

## Figures and Tables

**Table 1:** IC_50_ values of the most active compounds against *Rm*TIM and percentages of inhibition for human (*Hm*TIM) and rabbit (*Rb*TIM) at 100 µM.

Compound	Structure	IC_50_ (µM) *Rm*TIM	% Inhibition at 100 µM *Hm*TIM	% Inhibition at 100 µM *Rb*TIM
**98**	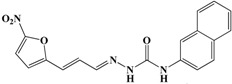	50 ± 6	14	21
**50**	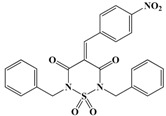	50 ± 10	0	0
**161**	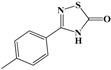	25 ± 3	28	0
**14**	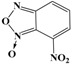	49 ± 1	0	0

**Table 2:** Inhibitory effects of compound **14** on *Rhipicephalus microplus* fully engorged females by immersion test. Larval weight *F* = 2.482. *p* value = 0.1639. Egg weight *F* = 5.254. *p* value = 0.0270, survival expressed in percentage.

Group	Total Oviposition	Eggs Total Weight (mg)	Larval Total Weight (mg)	Eclosion Rate
PBS	14/15	133 ± 33	107 ± 47	79%
DMSO	13/15	110 ± 45	73 ± 45	67%
C14-2000 ppm	13/15	123 ± 35	63 ± 33	51% *
C14-500 ppm	15/15	113 ± 47	60 ± 39	51% *

Dose-dependent response of adults (AIT) to compound **14**. The effects on the oviposition were calculated by the ratio of ticks that completed oviposition/total, total eggs, and larval weight (mg). The eclosion rate was calculated as described in Material and Methods. Standard deviation for each group was calculated. * represents statistical significance. Statistical analyses were performed using one-way ANOVA followed by Tukey’s test.

**Table 3:** Inhibitory effects of compound **14** on *Rhipicephalus microplus* fully engorged females. Injection of compound **14** into females treated during the artificial feeding procedure. Survival *F* = 126.9. *p* value < 0.0001.

Treatment	Oviposition/Total	Total Egg Weight (mg)	Total Larval Weight (mg)	Eclosion	Eclosion Rate
G1/PBS Buffer	YES 10/13	30.32 ± 16.39	12.6 ± 9.88	YES	43%
G2/DMSO	YES 3/13	48.67 ± 23.54	23.33 ± 16.92	YES	47%
G3/C14	NO 0/13	-	-	NO	-

Partially engorged female ticks were separated into homogeneous groups (G1, G2, and G3) and received a different treatment. The numbers are represented by the ratio of ticks that realized oviposition/total of ticks per group, and females that did not lay eggs were considered dead. Egg samples of each group were collected, subsequently weighed, and were represented by mass (milligram). ± represents the standard deviation from each group. - represents that oviposition did not occur. Statistical analyses were performed using one-way ANOVA followed by Tukey’s test.

## Supplementary Materials

The following are available online at http://www.mdpi.com/2306-7381/5/3/74/s1, Figure S1: Multiple alignment of the amino acid sequence among TIM from different ticks and B. Taurus, Figure S2: In vitro activity in BME26 embryonic cell from R. microplus, Table S1: Chemical structure of compounds used in primary screening.
