# Supplementary material for: Novel and Selective Rhipicephalus microplus Triosephosphate Isomerase Inhibitors with Acaricidal Activity
Source: Vet Sci. 2018 Aug 23;5(3):74. doi: 10.3390/vetsci5030074 (PMC6163981; doi:10.3390/vetsci5030074)
Supplement: Supplementary file 1 [file vetsci-05-00074-s001.pdf]

Table S1. Chemical structure of compounds used in primary screening.

Nº	Chemotype	*Rm*TIM %inhibition at 100µM	BME26 %inhibition at 100µM
**Benzofuroxans**			
1	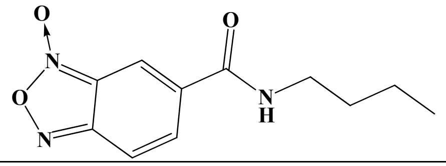	0	Nd
2	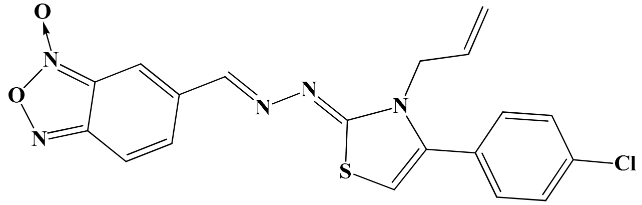	0	Nd
3	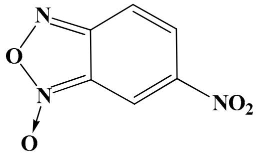	0	Nd
4	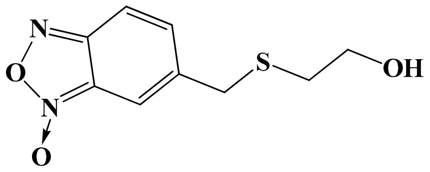	0	Nd

5	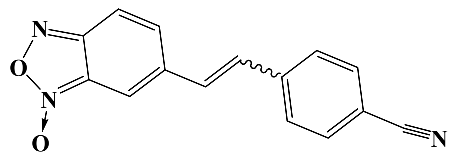	0	Nd
6	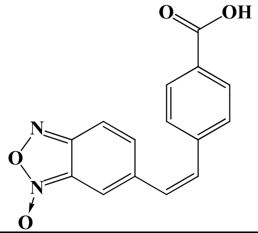	0	Nd
7	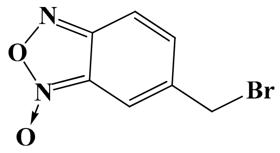	0	Nd
8	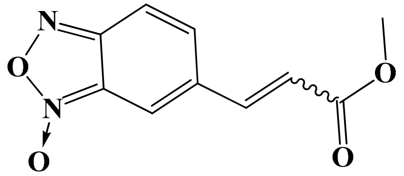	0	Nd
9	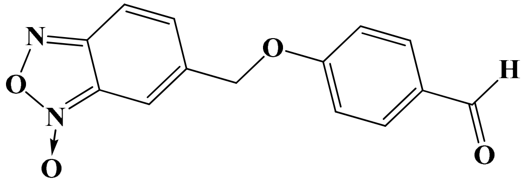	0	Nd
10	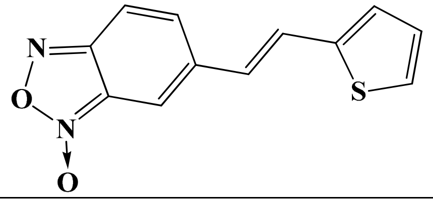	0	Nd

11	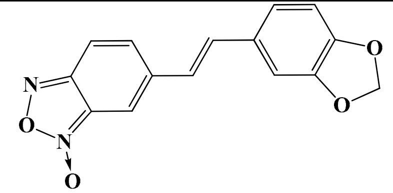	0	Nd
12	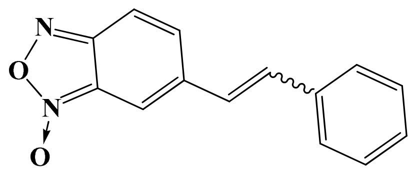	0	Nd
13	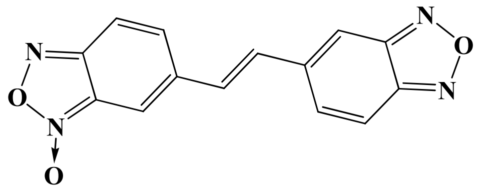	0	Nd
14	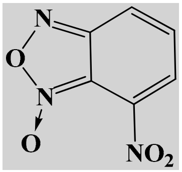	87	95
15	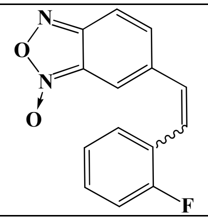	0	Nd
16	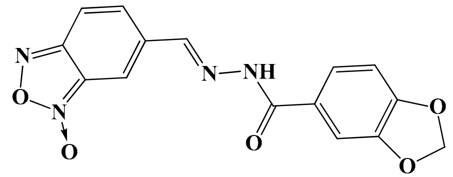	0	Nd

17	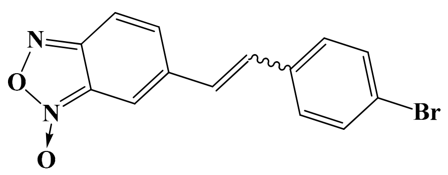	0	Nd
18	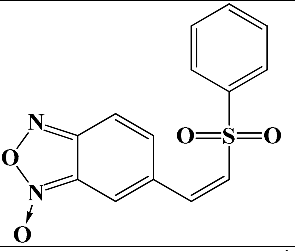	0	Nd
19	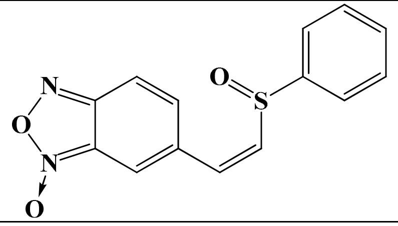	0	Nd
20	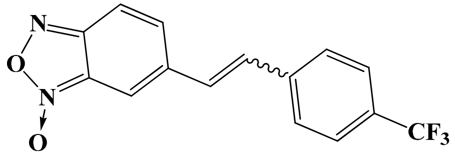	0	Nd
21	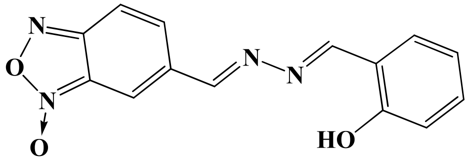	0	Nd

22	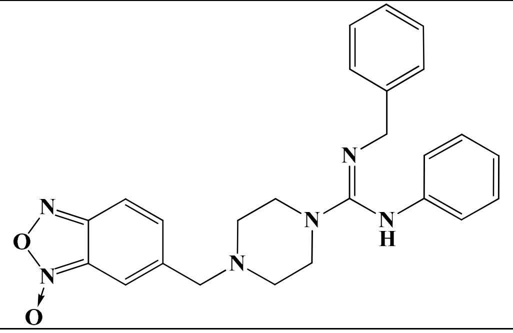	0	Nd
23	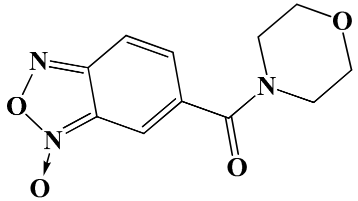	0	Nd
24	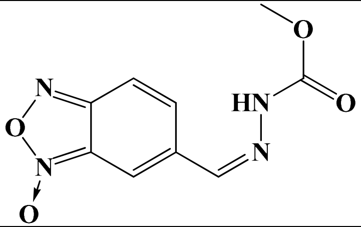	0	Nd
25	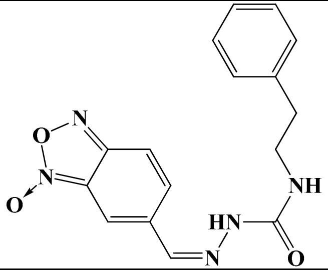	0	Nd
26	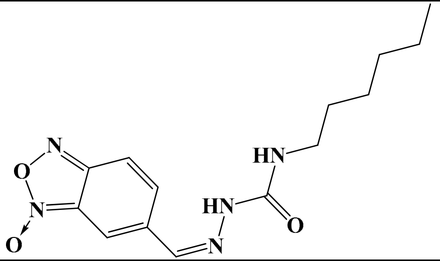	0	Nd

27	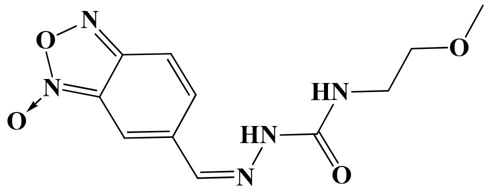	0	Nd
28	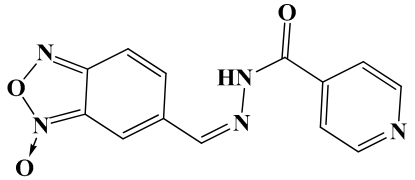	0	Nd
29	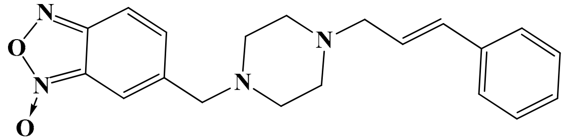	0	Nd
30	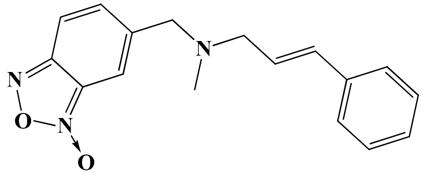	0	Nd
31	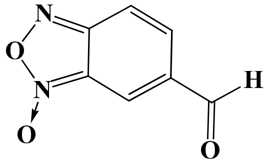	0	Nd
32	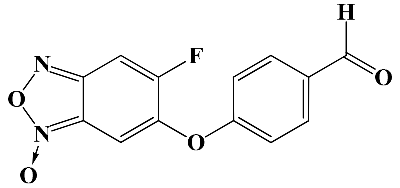	0	Nd

33	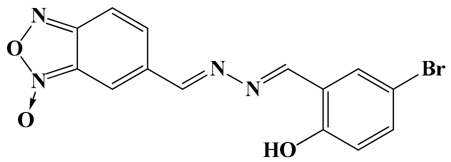	0	Nd
34	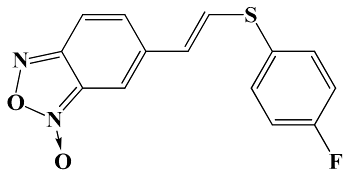	0	Nd
35	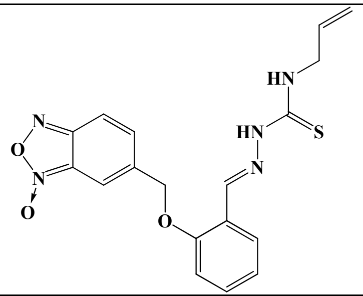	0	Nd
**Benzimidazoles**			
36	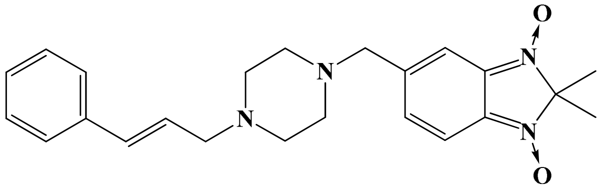	0	Nd
37	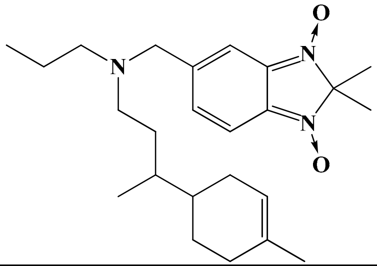	0	Nd

38	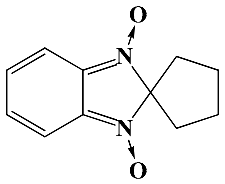	0	Nd
39	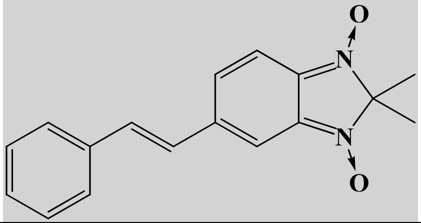	70	Nd
40	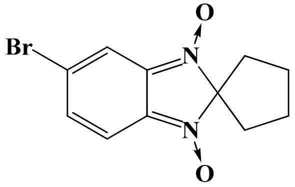	0	Nd
41	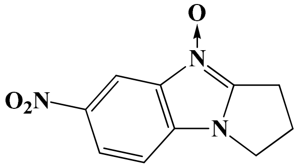	0	Nd
42	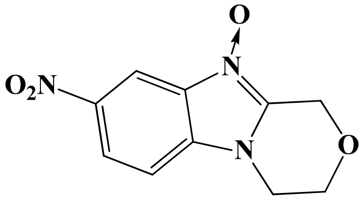	0	Nd
43	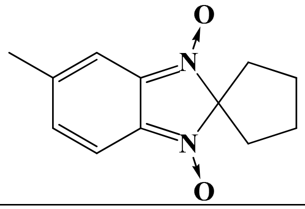	0	Nd

44	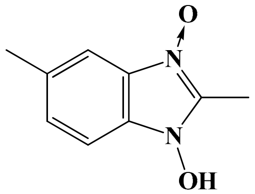	0	Nd
45	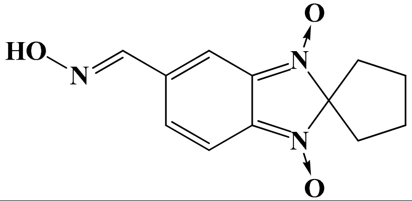	0	Nd
46	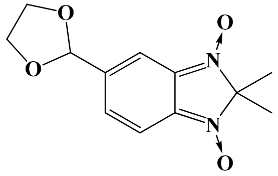	0	Nd
47	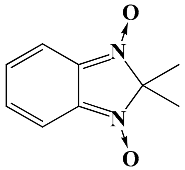	0	Nd
**Thiadiazines and synthetic precursors**			
48	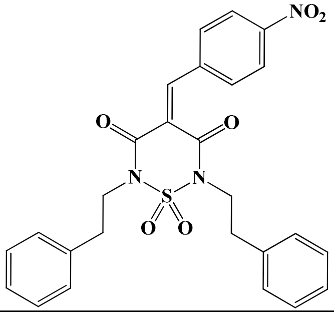	0	Nd

49	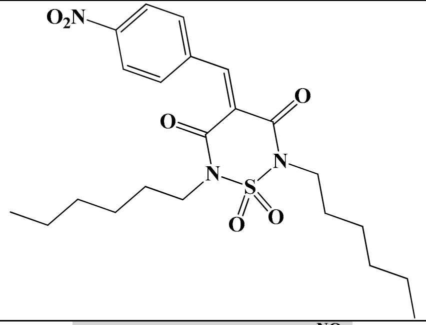	0	Nd
50	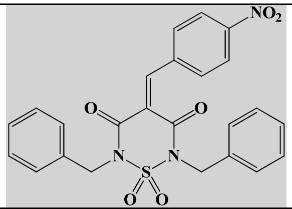	92	50
51	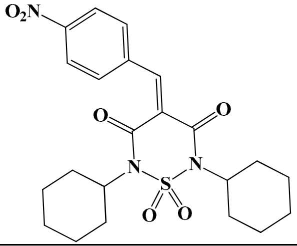	0	Nd
52	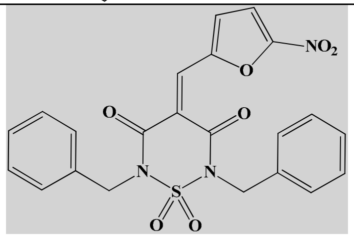	52	0
53	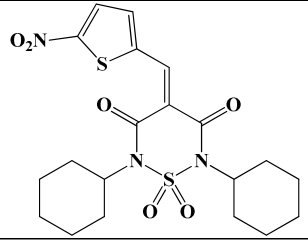	0	Nd

54	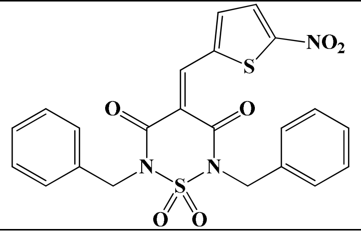	0	Nd
55	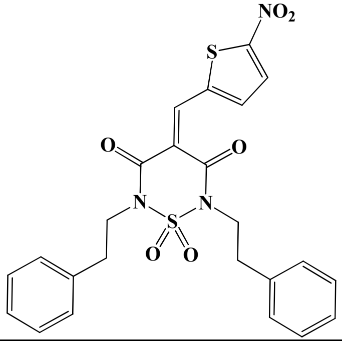	0	Nd
56	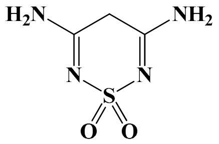	0	Nd
57	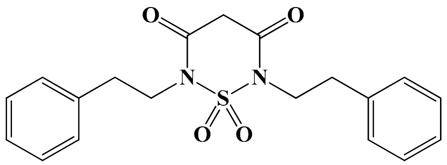	0	Nd
58	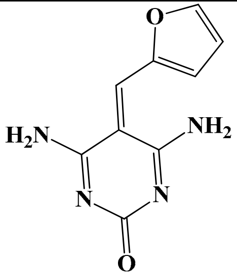	0	Nd

59	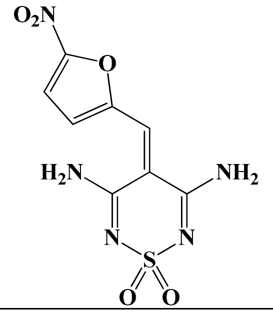	0	Nd
60	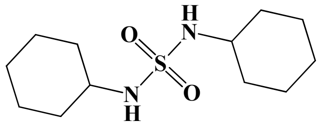	0	Nd
61	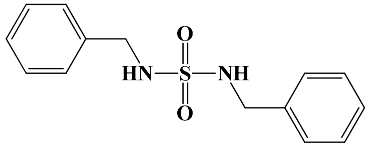	0	Nd
62	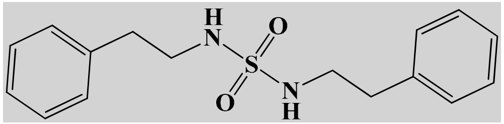	70	Nd
63	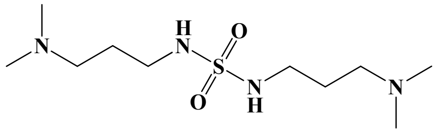	0	Nd
64	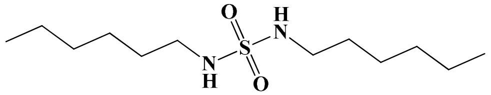	0	Nd

65	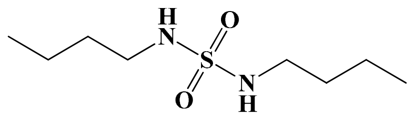	0	Nd
66	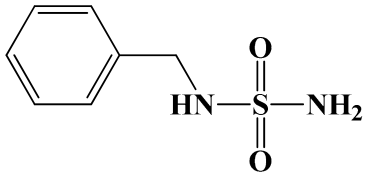	0	Nd
67	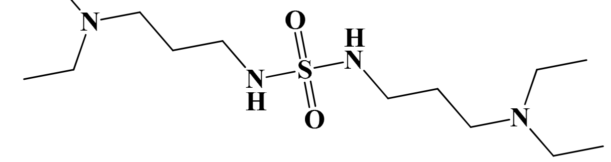	0	Nd
**Thiazolylidenehydrazines**			
68	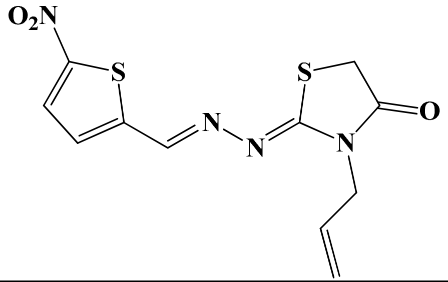	0	Nd
69	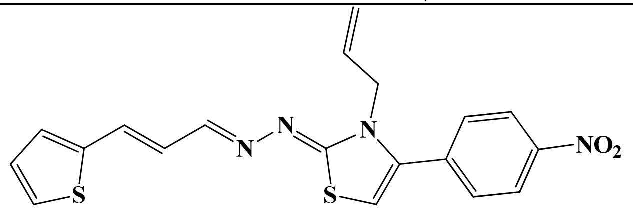	0	Nd

70	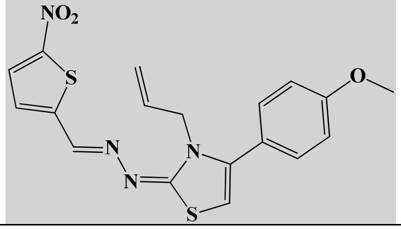	55	Nd
71	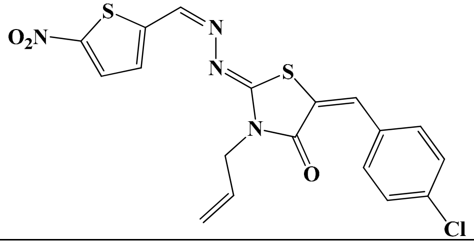	0	Nd
72	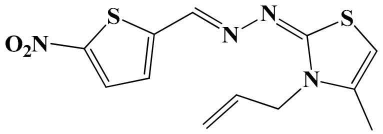	0	Nd
73	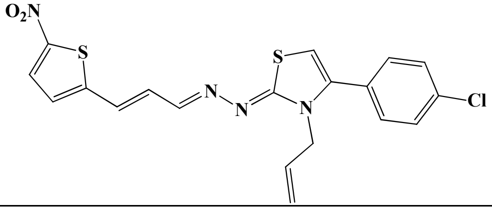	0	Nd
74	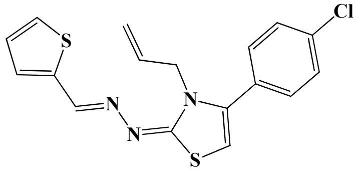	0	Nd
75	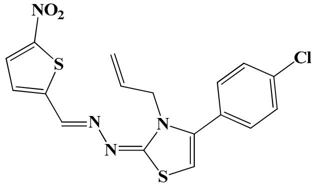	0	Nd

76	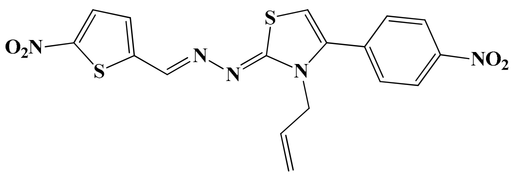	0	Nd
77	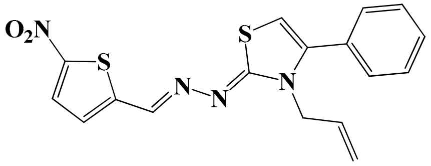	0	Nd
78	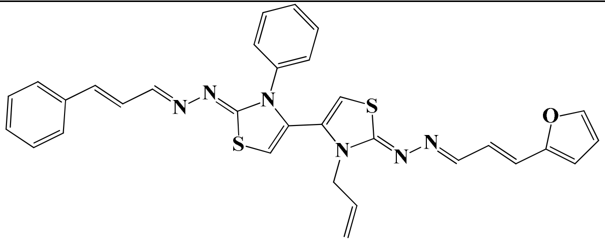	0	Nd
79	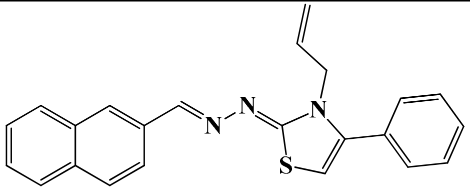	0	Nd
80	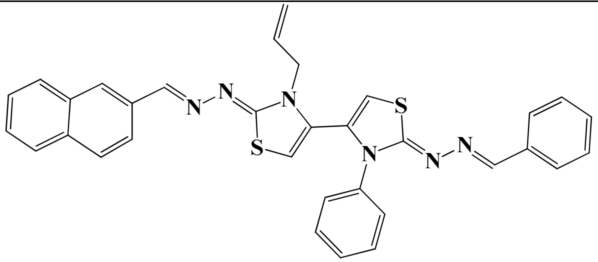	0	Nd

81	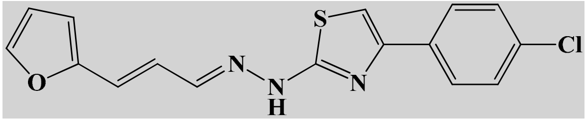	80	25
82	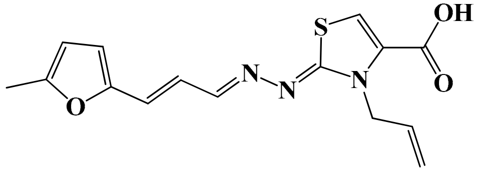	0	Nd
83	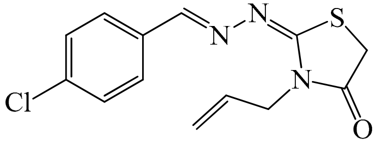	0	Nd
84	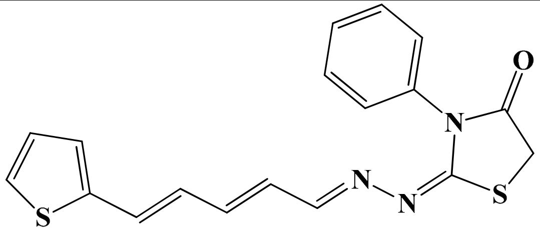	0	Nd
85	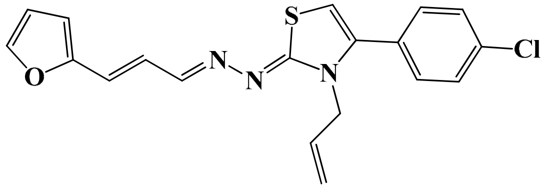	0	Nd

86	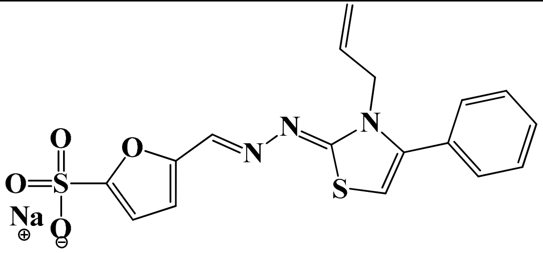	0	Nd
**Hydrazide derivatives**			
87	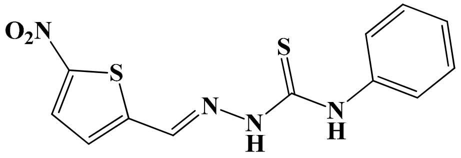	0	Nd
88	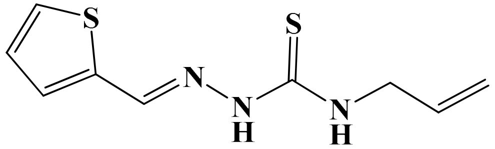	0	Nd
89	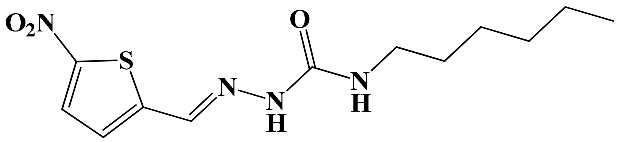	0	Nd
90	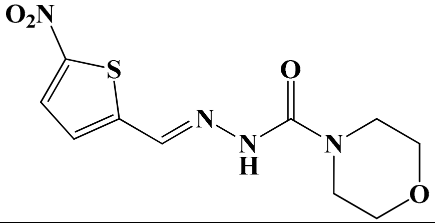	0	Nd

91	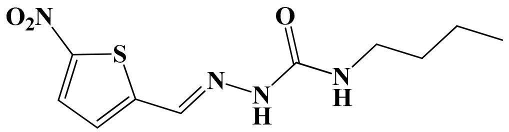	0	Nd
92	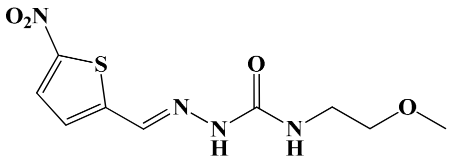	0	Nd
93	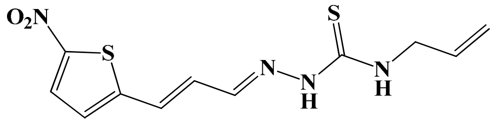	0	Nd
94	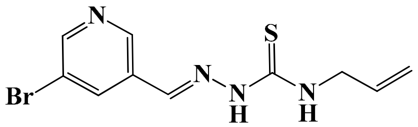	0	Nd
95	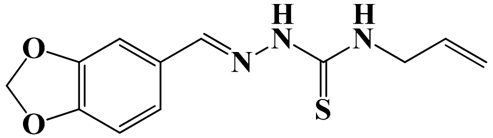	0	Nd
96	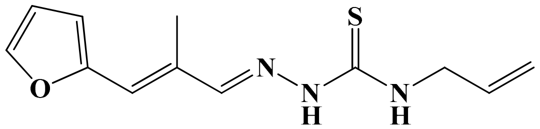	0	Nd

97	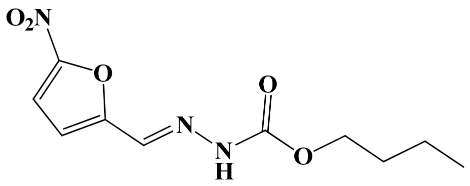	0	Nd
98	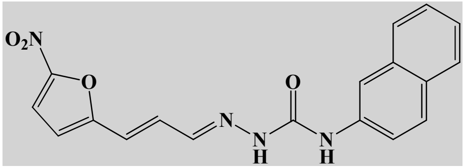	90	50
99	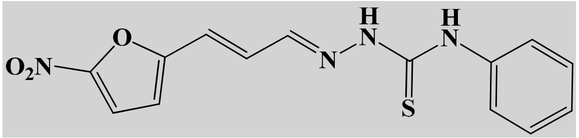	50	Nd
100	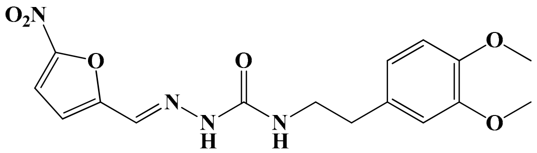	0	Nd
101	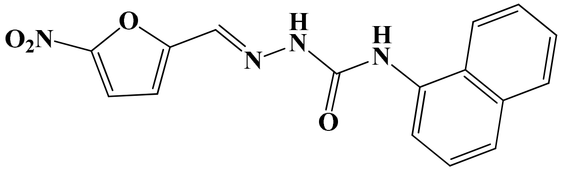	0	Nd
102	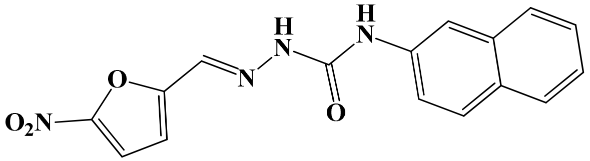	0	Nd

103	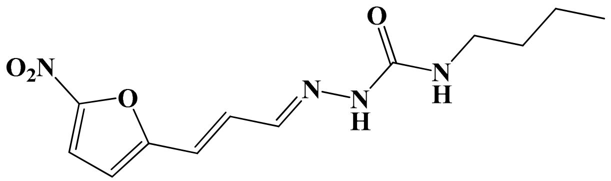	0	Nd
104	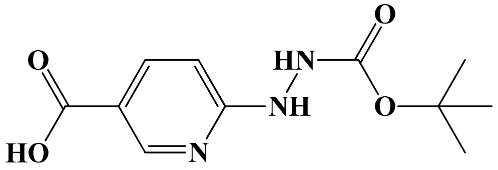	0	Nd
105	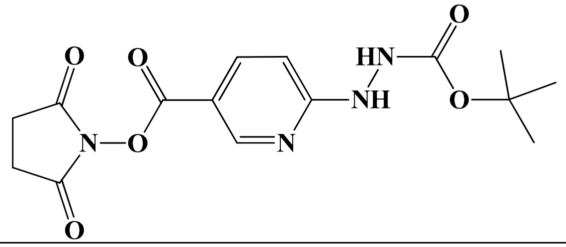	0	Nd
106	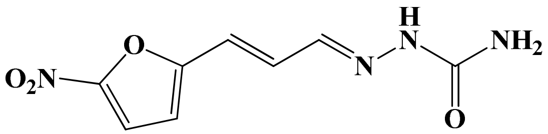	0	Nd
107	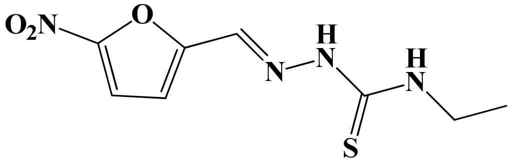	0	Nd
108	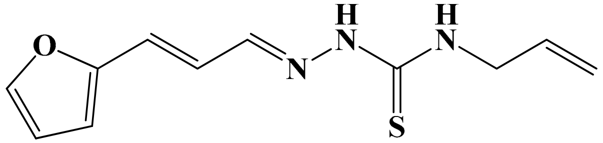	0	Nd

109	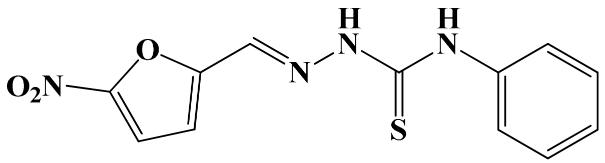	0	Nd
110	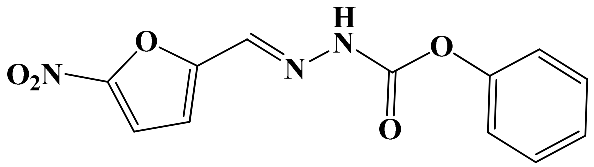	0	Nd
111	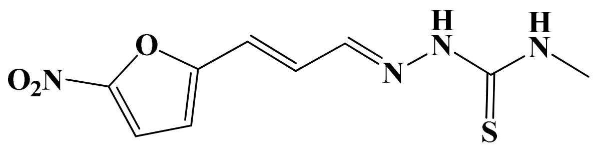	0	Nd
112	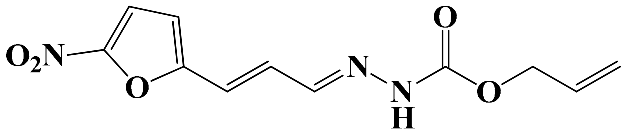	0	Nd
113	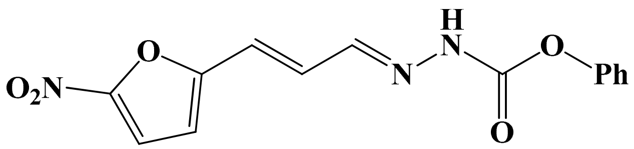	0	Nd
114	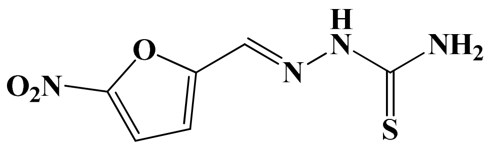	0	Nd

115	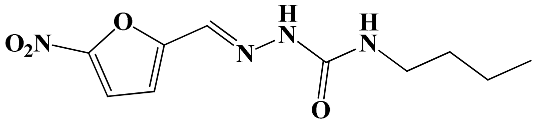	0	Nd
116	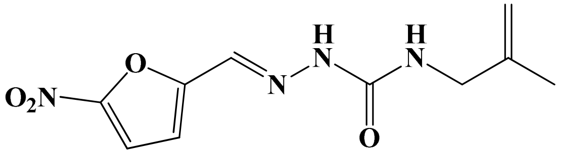	0	Nd
117	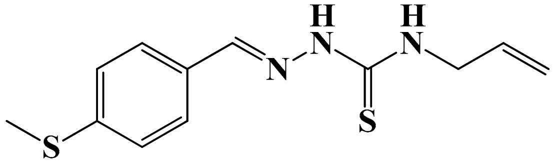	0	Nd
118	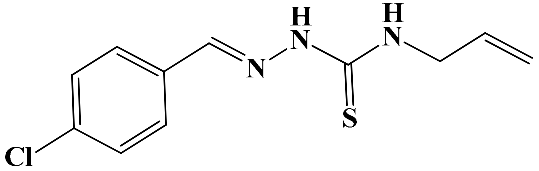	0	Nd
**Flavonoids**			
119	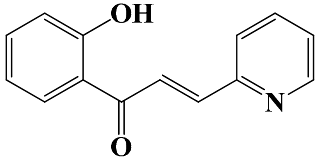	0	Nd
120	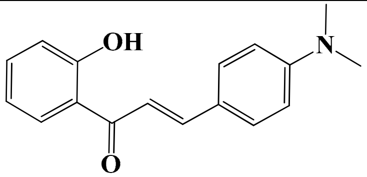	0	Nd

121	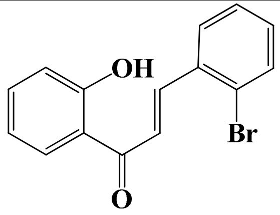	0	Nd
122	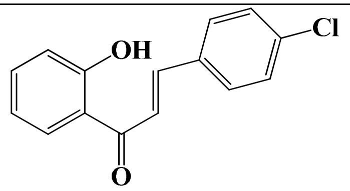	0	Nd
123	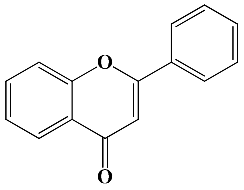	0	Nd
124	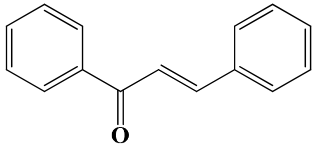	0	Nd
125	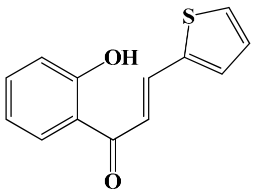	0	Nd
126	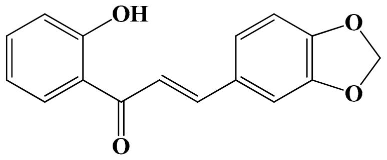	0	Nd

127	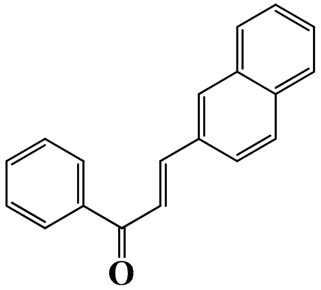	0	Nd
128	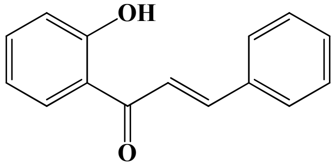	0	Nd
129	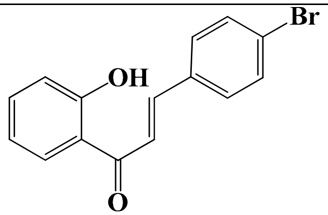	0	Nd
130	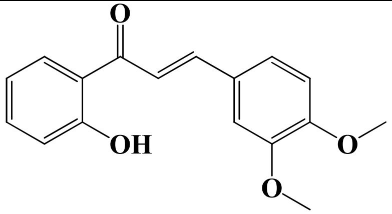	0	Nd
131	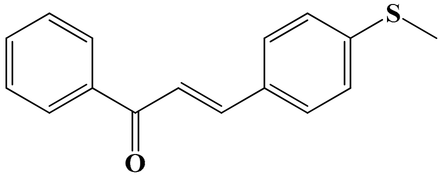	0	Nd
**Furoxans**			

132	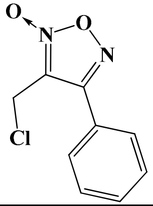	0	Nd
133	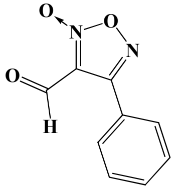	0	Nd
134	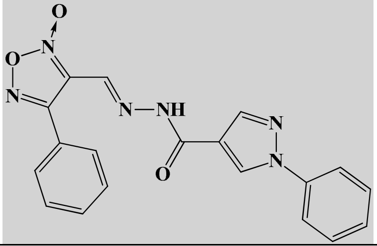	77	Nd
135	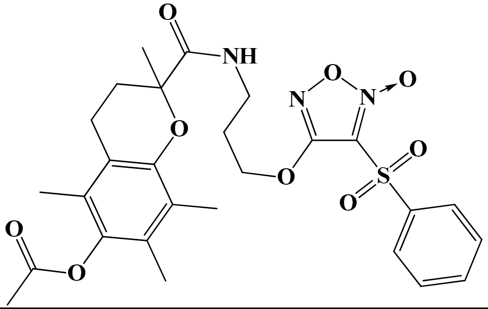	0	Nd
136	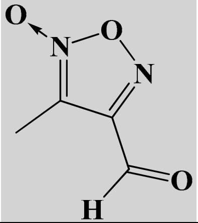	94	Nd

137	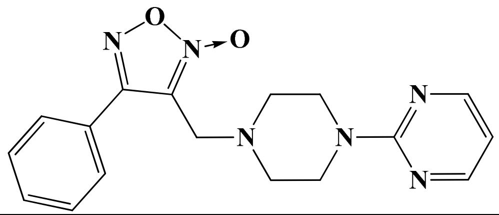	0	Nd
138	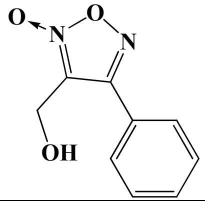	0	Nd
139	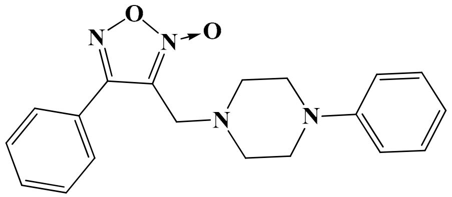	0	Nd
140	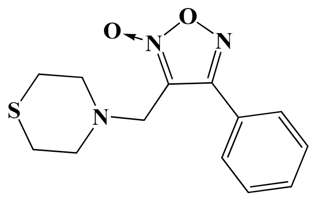	0	Nd
141	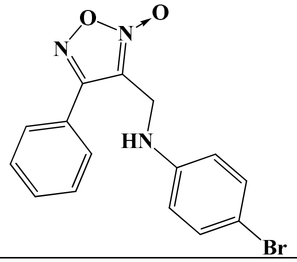	0	Nd

142	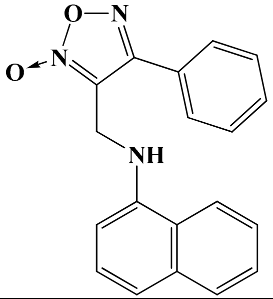	0	Nd
143	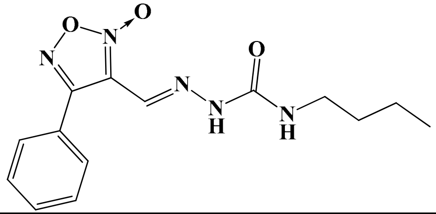	0	Nd
144	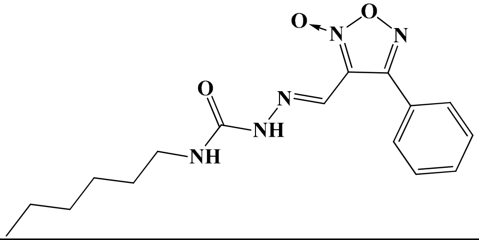	0	Nd
145	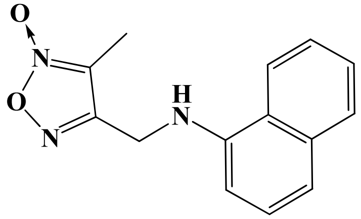	0	Nd
146	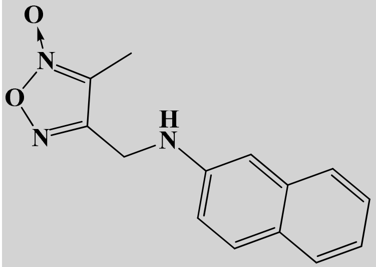	85	Nd

147	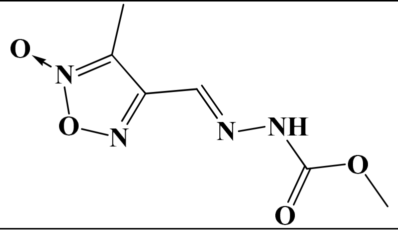	0	Nd
148	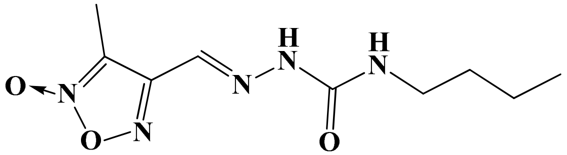	0	Nd
149	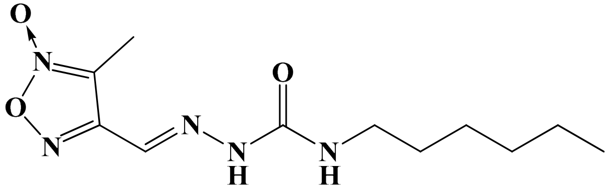	0	Nd
150	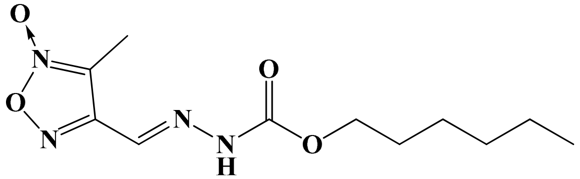	0	Nd
151	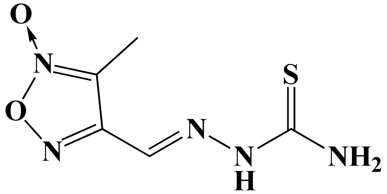	0	Nd
152	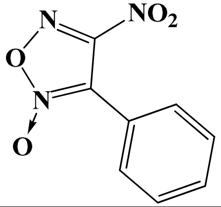	0	Nd

153	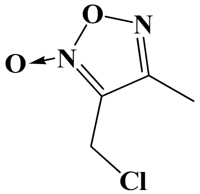	0	Nd
**Phenazines**			
154	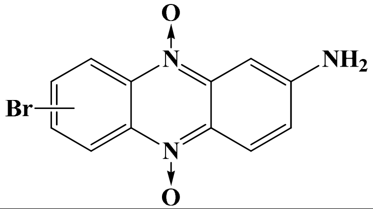	0	Nd
155	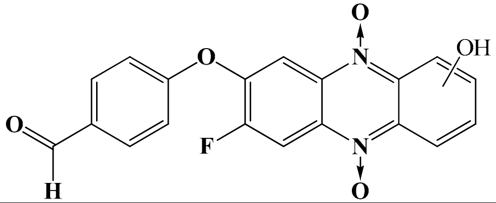	0	Nd
156	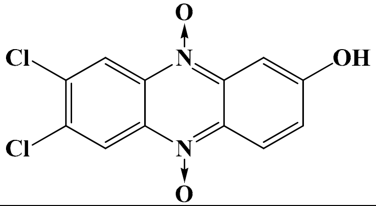	0	Nd
**Thiadiazoles**			
157	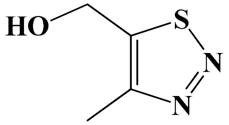	0	Nd

158	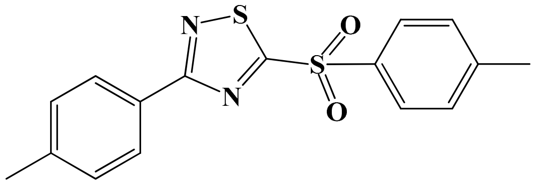	0	Nd
159	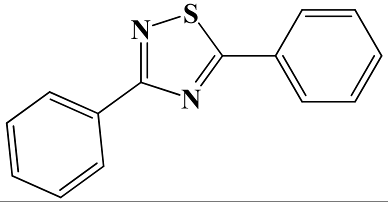	0	Nd
160	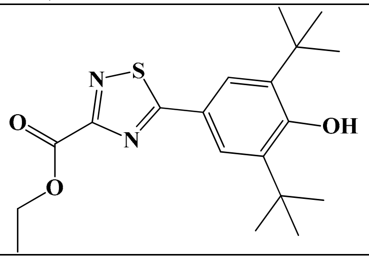	0	Nd
161	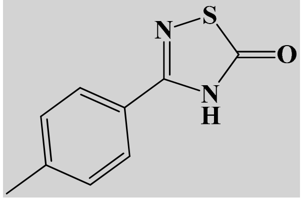	98	Nd
162	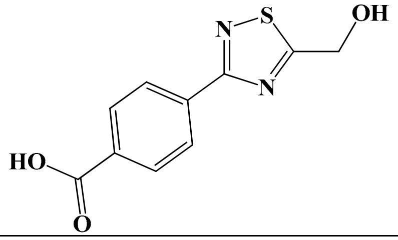	0	Nd
**Indazoles**			

163	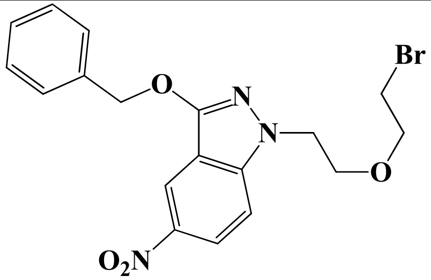	0	Nd
164	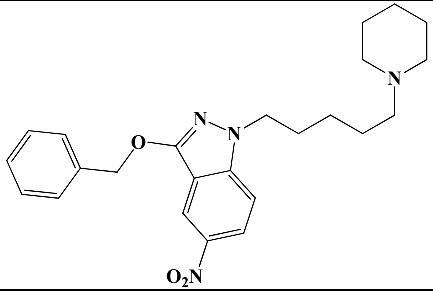	0	Nd
165	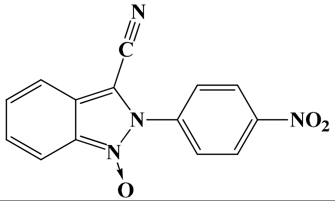	0	Nd
166	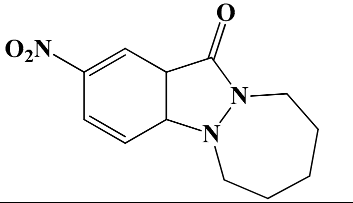	0	Nd
167	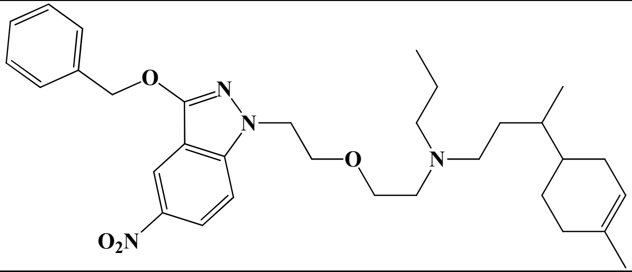	0	Nd

168	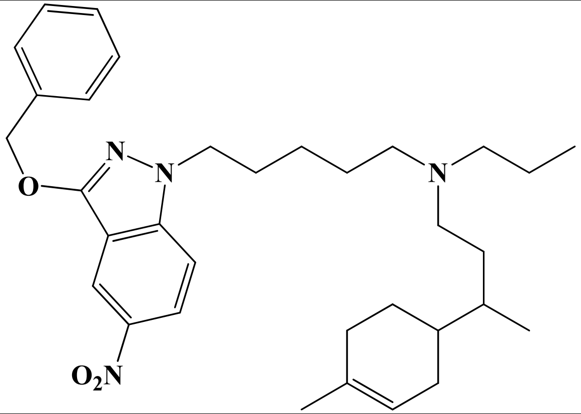	0	Nd
169	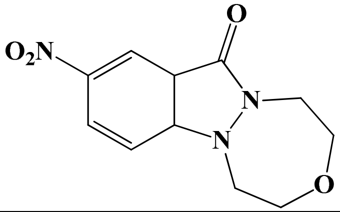	0	Nd
170	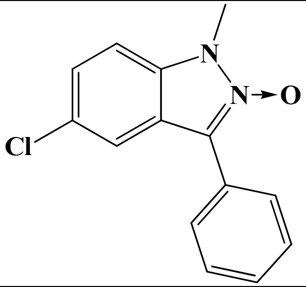	0	Nd
171	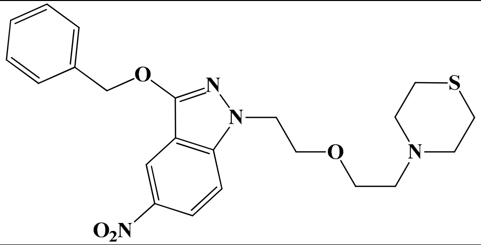	0	Nd

172	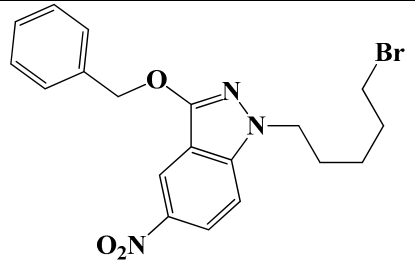	0	Nd
173	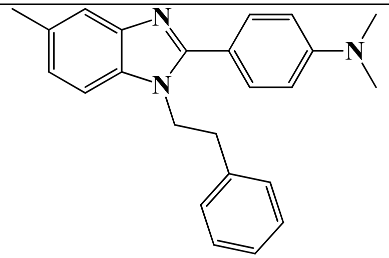	0	Nd
**Quinoxalines**			
174	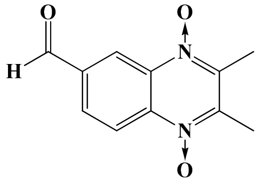	0	Nd
175	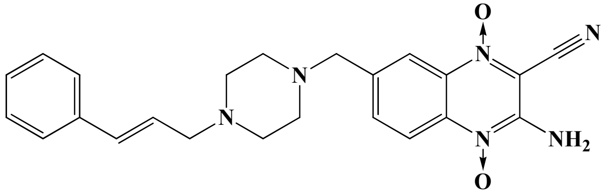	0	Nd
176	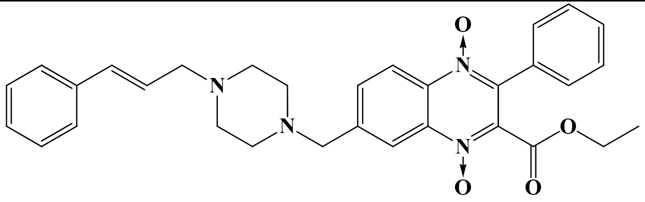	0	Nd

177	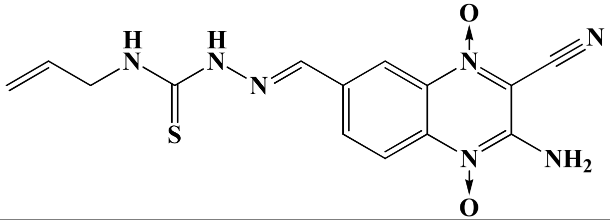	0	Nd
178	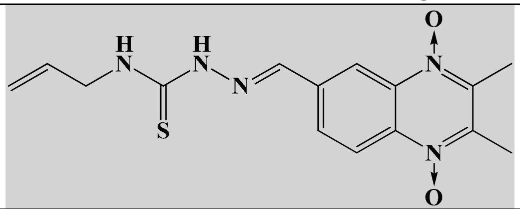	91	Nd
179	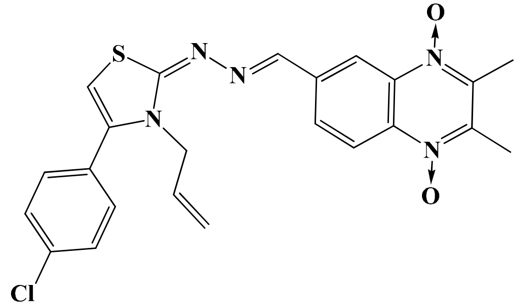	0	Nd
**Triazines**			
180	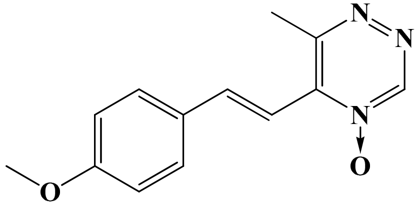	0	Nd
181	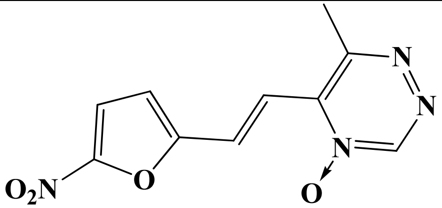	0	Nd

182	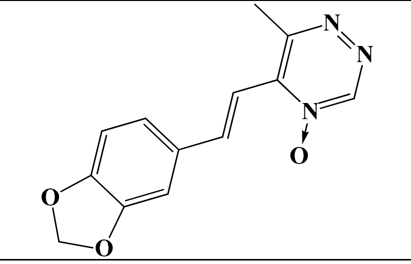	0	Nd
183	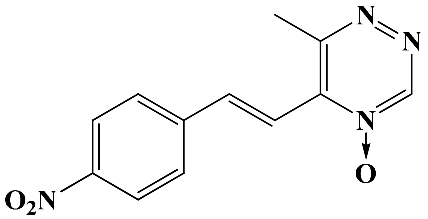	0	Nd
184	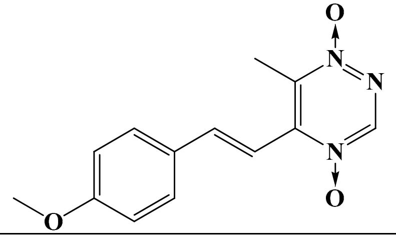	0	Nd
185	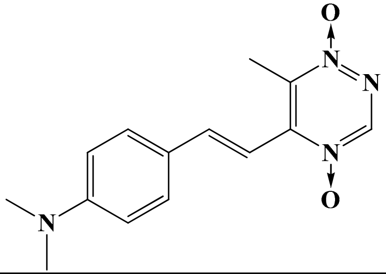	0	Nd
186	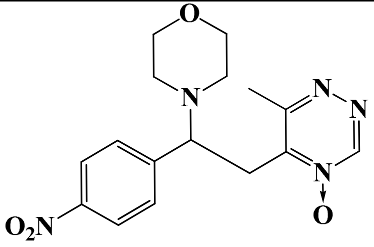	0	Nd

187	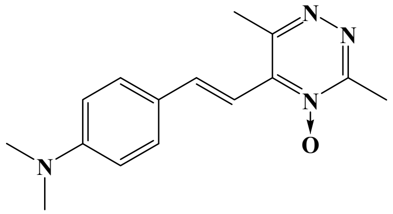	0	Nd
**Curcumin and analogues**			
188	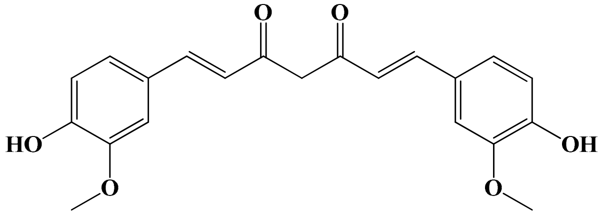	0	Nd
189	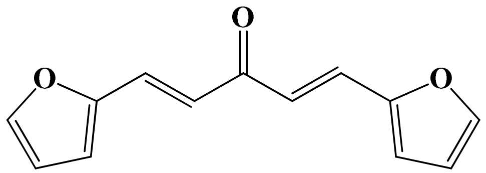	0	Nd
190	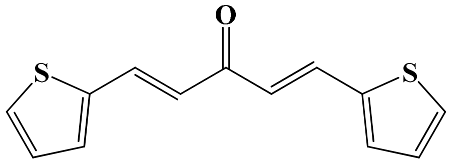	0	Nd
191	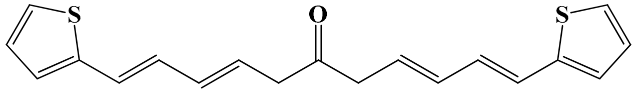	0	Nd

192	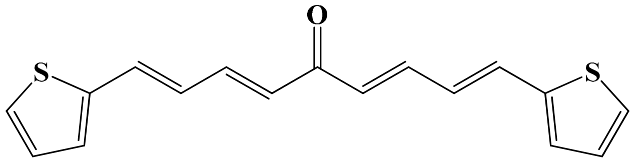	0	Nd
193	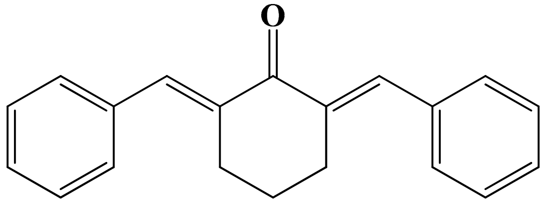	0	Nd
194	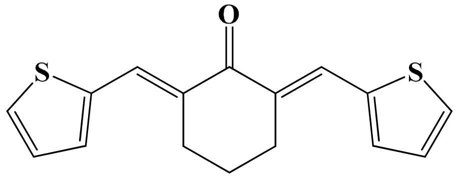	0	Nd
195	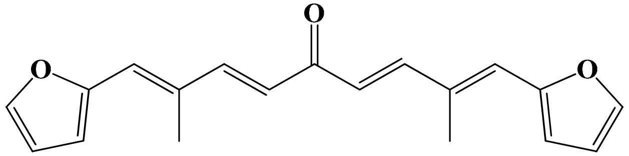	0	Nd
196	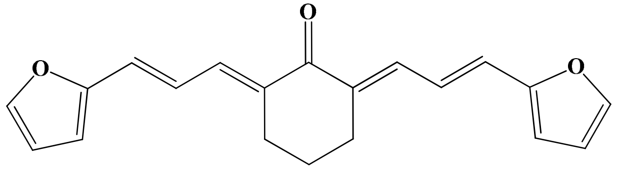	0	Nd
197	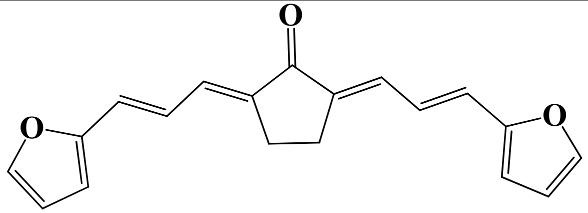	0	Nd

198	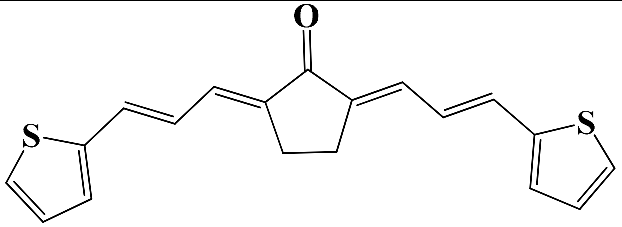	0	Nd
199	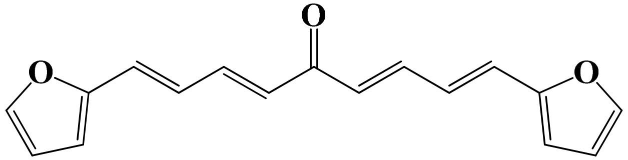	0	Nd
**No clustered structures**			
200	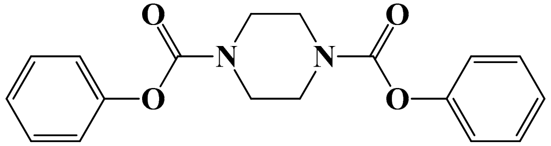	0	Nd
201	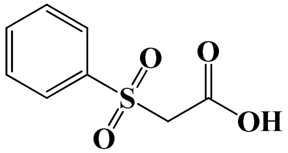	0	Nd
202	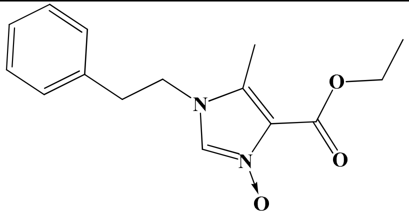	0	Nd

203	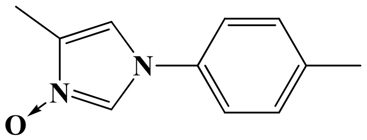	0	Nd
204	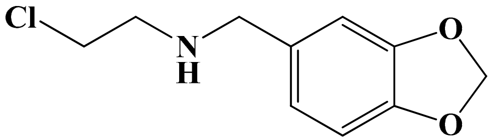	0	Nd
205	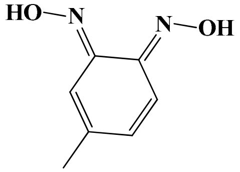	0	Nd
206	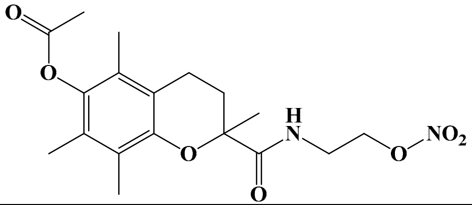	0	Nd
207	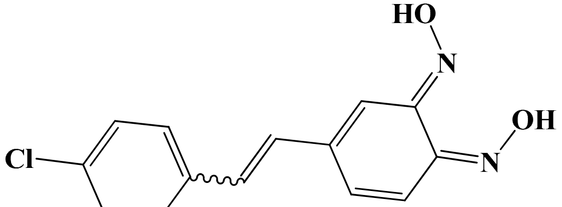	0	Nd
208	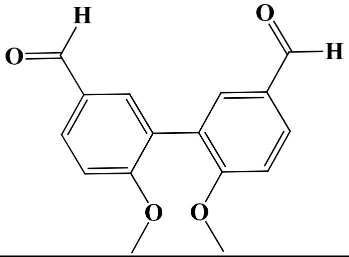	0	Nd

209	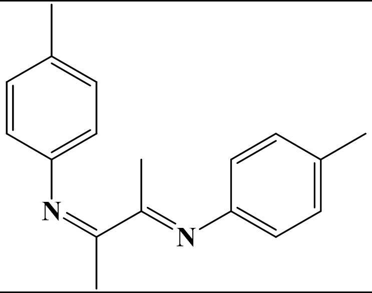	0	Nd
210	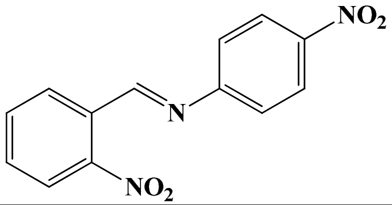	0	Nd
211	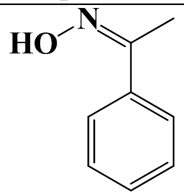	0	Nd
212	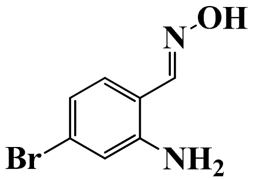	0	Nd
213	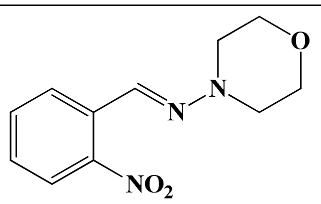	0	Nd

214	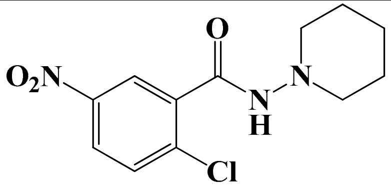	0	Nd
215	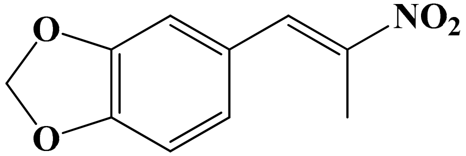	0	Nd
216	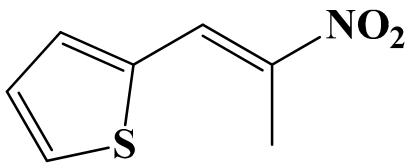	0	Nd
217	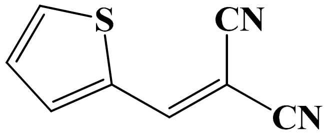	0	Nd
218	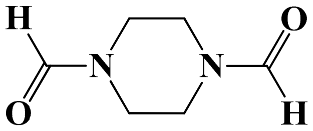	0	Nd
219	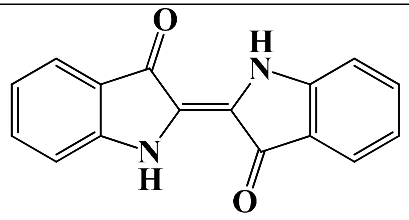	0	Nd

220	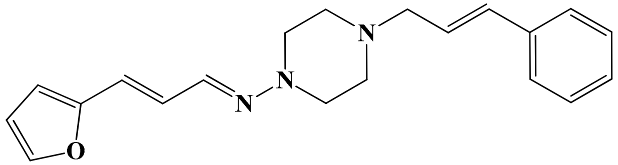	0	Nd
221	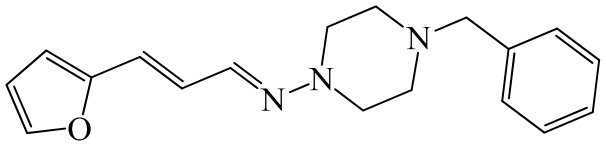	0	Nd
222	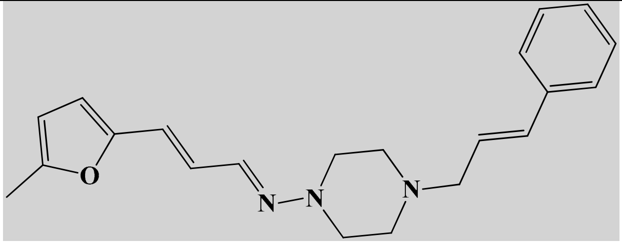	60	75
223	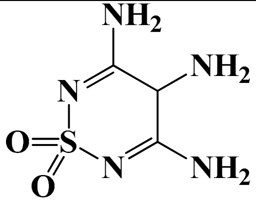	0	Nd
224	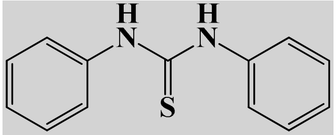	60	10
225	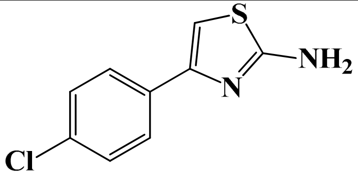	0	0

226	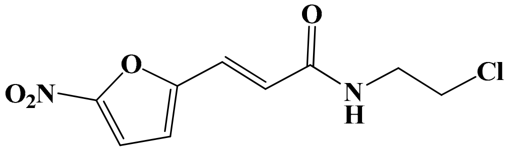	0	Nd
227	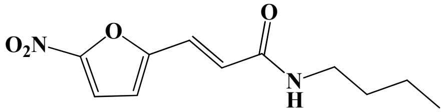	0	Nd

In gray are highlighted the best TIM inhibitors.

```
                              *        20         *        40         *
B. taurus    : MAPSRKFFVGGNWKMNGRKNNLGELINTLNAAKVPADTEVVCAPPTAYID :  50
A.cajennense: --MARRFVVGGNWKMNGNKNSIRDICNALKGANLDPNTEVVIGCPAPYLD :  48
A. maculatum: --MARRFVVGGNWKMNGNKNSIRDICNTLKGASLDPNTEVVIGCPAPYLD :  48
A. parvum    : --MARRFVVGGNWKMNGNKNSIRDICNTLKGASLDPNTEVVIGCPAPYLD :  48
A. triste    : --MARRFVVGGNWKMNGNKNSIRDICNTLKGASLDPNTEVVIGCPAPYLD :  48
R. microplus: -MAARRFCVGGNWKMHGSKNSIRDICNTLKGASLDPNVEVIVACPAPYLD :  49
H. excavatum  -MATRRFCVGGNWKMNGSKNSIRDICNTLKGACLDPSTEVIIACPAPYLD :  49
I. ricinus   : -MSGRKFCVGGNWKMNGNKGSIKEICDMLKTAKLDPNTEVILGCPAPYLD :  49
I scapularis: -MSGRKFCVGGNWKMNGNKSSIKEICDMLKTAKLDPNTEVVLGCPAPYLD :  49
R.pulchellus: -MAARRFCVGGNWKMHGSKNSIRDICNNLKSSCLDPNVEVIIACPAPYLD :  49
                       60         *        80         *       100
B. taurus    : FARQKLDPKIAVAAQNCYKVANGAFTGEISPGMIKILGATWVVLGHSERR : 100
A.cajennense: YCRSLLPPSVALAAQNCYKVEKGAFTGEISPAMIKICGGQWVILGHSERR :  98
A. maculatum: YCRSLLPPSVALAAQNCYKVEKGAFTGEISPAMIKICGGQWVILGHSERR :  98
A. parvum    : YCRSLLPPSVALAAQNCYKVEKGAFTGEISPAMIKICGGQWVILGHSERR :  98
A. triste    : YCRSLLPPSVALAAQNCYKVEKGAFTGEISPAMIKICGGQWVILGHSERR :  98
R. microplus: YCRSLLPPSVALAAQNCYKVEQGAFTGEISPGMIKICGGQWVILGHSERR :  99
H. excavatum  YCRSLLPPSVALAAQNCYKVEKGAFTGEISPGMIKICGGQWVILGHSERR :  99
I. ricinus   : YVRRILPAAIAVSAQNCYKVEKGAFTGEISPAMIKICGATWVILGHSERR :  99
I scapularis: YVRRILPAAIAVSAQNCYKVEKGAFTGEISPAMIKICGATWVILGHSERR :  99
R.pulchellus: YCRSLLPPSVALSAENCYKVEQGAFTGEISAGMIKICGGTWVILGHSERR :  99
                        *       120         *       140         *
B. taurus    : HVFGESDELIGQKVAHALAEGLGVIACIGEKLDEREAGITEKVVFEQTKV : 150
A.cajennense: HVFKETDELIGEKVKHALESGLNVIACIGELLEEREANKTEEVCYRQTKA : 148
A. maculatum: HVFKETDELIGEKVKHALESGLGVIACIGELLEEREANKTEEVCYCQMKA : 148
A. parvum    : HVFKETDELIGEKVKHALDSGLNVIACIGELLEERESNKTEEVCYRQTKA : 148
A. triste    : HVFKETDELIGEKVKHALESGLGVIACIGELLEEREANKTEEVCYCQMKA : 148
R. microplus: HVFKEDDVLIGEKIKHALESGLNVIACIGELLEDREAGRTEEVCFRQIKH : 149
H. excavatum  HIFKETDELIGEKVKHALESGLGVIACIGELLEERESNKTEEVCFRQTKA : 149
I. ricinus   : NVFKESDELIGDKVHHALESGLNVIACIGELLEEREAGKTEEVVYRQTAA : 149
I scapularis: NVFKESDELIGDKVHHALESGLNVIACIGELLEEREAGKTEEVVYRQTAA : 149
R.pulchellus: HIFKEDDVLIGEKIKHALGTGLNVIACIGELLEDREAGRTEDVLFGQMKY : 149
                    160         *       180         *       200
B. taurus    : IADNVKDWSKVVLAYEPVWAIGTGKTATPQQAQEVHEKLRGWLKSNVSDA : 200
A.cajennense: IAANVKDWGKVVIAYEPVWAIGTGKTATPDQAQEIHAKVRNWISTNVSPD : 198
A. maculatum: IAANVKDWSKVVIAYEPVWAIGTGKTATPDQAQEIHAKVRSWLSTNVSPD : 198
A. parvum    : IAGNVKDWGKVVIAYEPVWAIGTGKTATPDQAQEIHAKVRNWISTNVSPD : 198
A. triste    : IAANVKDWSKVVIAYEPVWAIGTGKTATPDQAQEIHAKVRSWLSTNVSPD : 198
R. microplus: IASNVKDWSKVVIAYEPVWAIGTGKTATPDQAQEVHSKVRNWLSTNVSAD : 199
H. excavatum  IASNVKDWSKVVIAYEPVWAIGTGKTATPDQAQEIHSKVRQWLSSNVSAD : 199
I. ricinus   : IAAKVKDWDRVVLAYEPVWAIGTGKTASPEQAQEVHAQLRQWLSKNVSPD : 199
I scapularis: IAAKVTDWNRVVLAYEPVWAIGTGKTASPEQA-EVHAQLRQWLSKNVSPD : 198
R.pulchellus: IAPNVSDWNKMIIAYEPVWAIGTGKTATPDQAQEVHSKVRNWLATNVSPD : 199
                        *       220         *       240         *
B. taurus    : VAQSARIIYGGSVTGATCKELASQPDVDGFLVGGASLKPEFVDIINAKQ- : 249
A.cajennense: VAAKVRIQYGGSVTAANCKELSRKSDIDGFLVGGASLKPEFVDIINARQ- : 247
A. maculatum: VAAKVRIQYGGSVTAANCKELARKPDIDGFLVGGASLKPEFVQIINARQ- : 247
A. parvum    : VAAKVRIQYGGSVTAANCKELARKPDIDGFLVGGASLKPEFVEIINARQ- : 247
A. triste    : VAAKVRIQYGGSVTAANCKELARKPDIDGFLVGGASLKPEFVQIINARQ- : 247
R. microplus: VASKVRIQYGGSVNAGNCKELGRKPDIDGFLVGGASLKPEFVQIINAMQG : 249
H. excavatum  VASKVRIQYGGSVNAGNCKELARKPDIDGFLVGGASLKPEFVQIINARQG : 249
I. ricinus   : VAKKVRIQYGGSVTAANCRELAKKPDVDGFLVGGASLKPEFVEIINARQ- : 248
I scapularis: VAKKVRIQYGGSVTAANCQELAKKPDVDGFLVGGASLKPEFVEIINARQ- : 247
R.pulchellus: VAAKIRMQYGGSVNAGNCRELARKPDIDGFLVGGASLKPEFVQIINCHQG : 249
```

Figure S1: Multiple alignment of the amino acid sequence among TIM from different ticks and *B. Taurus.*

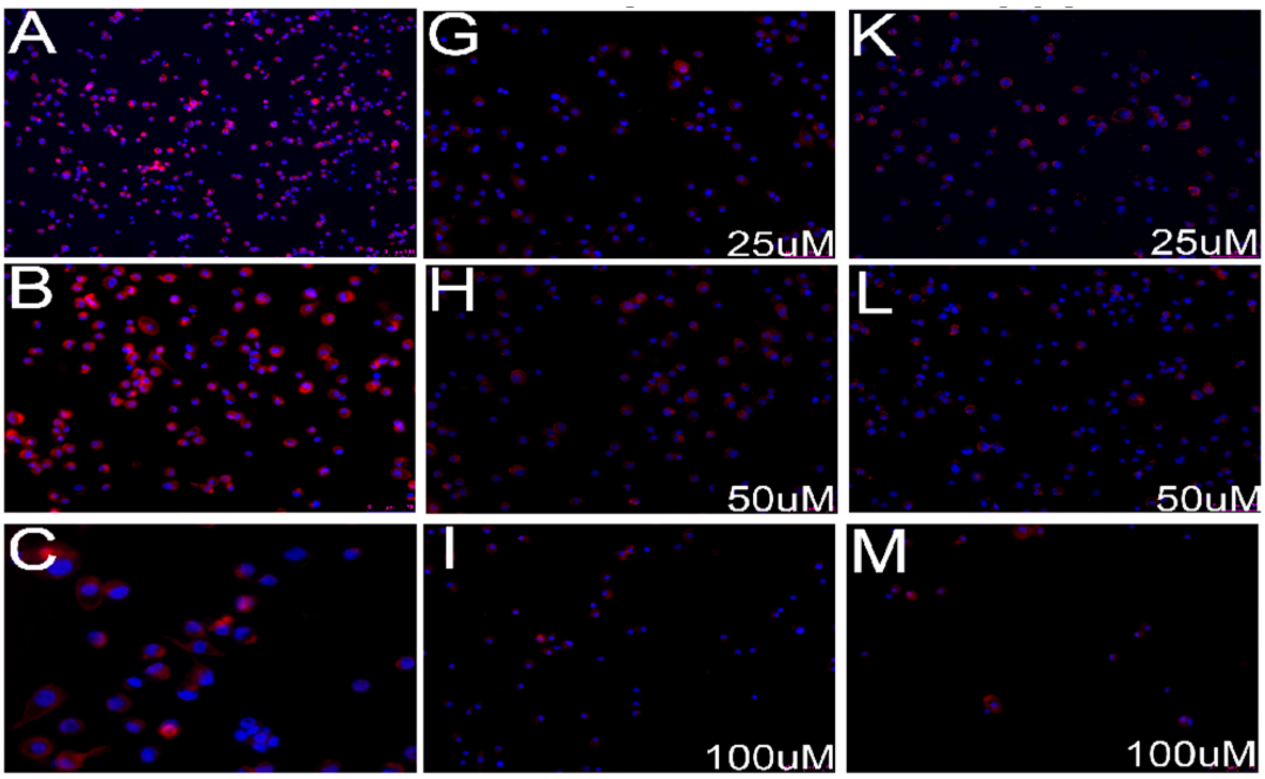

Figure S2: *In vitro* activity in BME26 embryonic cell from *R. microplus*.
